# Histone Loaders CAF1 and HIRA Restrict Epstein-Barr Virus B-Cell Lytic Reactivation

**DOI:** 10.1128/mBio.01063-20

**Published:** 2020-10-27

**Authors:** Yuchen Zhang, Chang Jiang, Stephen J. Trudeau, Yohei Narita, Bo Zhao, Mingxiang Teng, Rui Guo, Benjamin E. Gewurz

**Affiliations:** a Division of Infectious Disease, Department of Medicine, Brigham and Women’s Hospital, Harvard Medical School, Boston, Massachusetts, USA; b Sun Yat-sen University Cancer Center, State Key Laboratory of Oncology in South China, Guangzhou, China; c Department of Microbiology, Harvard Medical School, Boston, Massachusetts, USA; d Broad Institute of Harvard and MIT, Cambridge, Massachusetts, USA; e Harvard Graduate Program in Virology, Harvard University, Boston, Massachusetts, USA; f Department of Biostatistics and Bioinformatics, H. Lee Moffitt Cancer Center and Research Institute, Tampa, Florida, USA; University of North Carolina, Chapel Hill

**Keywords:** latency, lytic reactivation, histone chaperone, histone loader, epigenetic, restriction factor, histone methylation, CRISPR, interferon-stimulated gene, chromatin, tumor virus, gammaherpesvirus

## Abstract

Epstein-Barr virus (EBV) infects 95% of adults worldwide and causes infectious mononucleosis. EBV is associated with endemic Burkitt lymphoma, Hodgkin lymphoma, posttransplant lymphomas, nasopharyngeal and gastric carcinomas. In these cancers and in most infected B-cells, EBV maintains a state of latency, where nearly 80 lytic cycle antigens are epigenetically suppressed. To gain insights into host epigenetic factors necessary for EBV latency, we recently performed a human genome-wide CRISPR screen that identified the chromatin assembly factor CAF1 as a putative Burkitt latency maintenance factor. CAF1 loads histones H3 and H4 onto newly synthesized host DNA, though its roles in EBV genome chromatin assembly are uncharacterized. Here, we found that CAF1 depletion triggered lytic reactivation and virion secretion from Burkitt cells, despite also strongly inducing interferon-stimulated genes. CAF1 perturbation diminished occupancy of histones 3.1 and 3.3 and of repressive histone 3 lysine 9 and 27 trimethyl (H3K9me3 and H3K27me3) marks at multiple viral genome lytic cycle regulatory elements. Suggestive of an early role in establishment of latency, EBV strongly upregulated CAF1 expression in newly infected primary human B-cells prior to the first mitosis, and histone 3.1 and 3.3 were loaded on the EBV genome by this time point. Knockout of CAF1 subunit CHAF1B impaired establishment of latency in newly EBV-infected Burkitt cells. A nonredundant latency maintenance role was also identified for the DNA synthesis-independent histone 3.3 loader histone regulatory homologue A (HIRA). Since EBV latency also requires histone chaperones alpha thalassemia/mental retardation syndrome X-linked chromatin remodeler (ATRX) and death domain-associated protein (DAXX), EBV coopts multiple host histone pathways to maintain latency, and these are potential targets for lytic induction therapeutic approaches.

## Abstract (precis)

Epstein-Barr virus (EBV) was discovered as the first human tumor virus in endemic Burkitt lymphoma, the most common childhood cancer in sub-Saharan Africa. In Burkitt lymphoma and in 200,000 EBV-associated cancers per year, epigenetic mechanisms maintain viral latency, during which lytic cycle factors are silenced. This property complicated EBV’s discovery and facilitates tumor immunoevasion. DNA methylation and chromatin-based mechanisms contribute to lytic gene silencing. Here, we identified histone chaperones CAF1 and HIRA, which have key roles in host DNA replication-dependent and replication-independent pathways, respectively, as important for EBV latency. EBV strongly upregulates CAF1 in newly infected B-cells, where viral genomes acquire histone 3.1 and 3.3 variants prior to the first mitosis. Since histone chaperones ATRX and DAXX also function in maintenance of EBV latency, our results suggest that EBV coopts multiple histone pathways to reprogram viral genomes and highlight targets for lytic induction therapeutic strategies.

## INTRODUCTION

The gammaherpesvirus Epstein-Barr virus (EBV) persistently infects nearly 95% of adults worldwide (1). EBV is the etiological agent of infectious mononucleosis and is also causally associated with multiple human cancers, including endemic Burkitt lymphoma (eBL), Hodgkin lymphoma, posttransplant lymphoproliferative disease, HIV-associated lymphomas, nasopharyngeal carcinoma, and gastric carcinoma (2). Tumor cells contain multiple copies of chromatinized, nonintegrated, double-stranded DNA EBV genomes, where incompletely defined epigenetic pathways maintain a state of viral latency and in which most cells do not produce infectious virus.

EBV initiates lifelong infection by translocating across the tonsillar epithelium to colonize the B-cell compartment (3, 4). Virions deliver unchromatinized, encapsidated, linear EBV genomes to newly infected cells, which traffic to the nucleus. Upon nuclear entry, incoming genomes are circularized by host DNA ligases and chromatinized (1, 5, 6).

The EBV genome encodes nearly 80 proteins, most of which are highly immunogenic. To evade immune detection, EBV switches between latent and lytic genome programs, a hallmark of herpesvirus infection. Multiple layers of epigenetic regulation enable EBV to establish latency in newly infected B-cells, in which a small number of virus-encoded proteins and viral noncoding RNAs (ncRNAs) reprogram the metabolism, growth, and survival pathways of infected cells (7–9). Within 3 days of infection, quiescent B-cells are reprogramed to become rapidly growing lymphoblasts that divide as frequently as every 8 h (10–13).

According to the germinal center model (3), EBV-infected B-cells navigate the B-cell compartment to differentiate into memory cells, the reservoir for persistent EBV infection. To accomplish this, a series of EBV latency programs are used in which combinations of Epstein-Barr nuclear antigens (EBNA), latent membrane proteins (LMP), and ncRNAs are expressed (1). Memory cells exhibit the latency I program, in which Epstein-Barr nuclear antigen 1 (EBNA1) is the only EBV-encoded protein expressed. EBNA1 tethers the EBV genome to host chromosomes and has key roles in the propagation of viral genomes to daughter cells. EBNA1 is poorly immunogenic, facilitating immune escape of latency I cells.

Plasma cell differentiation is a trigger for EBV lytic reactivation. Induction of two viral immediate early gene transcription factors, BZLF1 and BRLF1, induces nearly 30 early genes important for production of lytic genomes (10, 14, 15). How these newly synthesized EBV genomes evade chromatinization by host histone loaders, including the heterotrimeric chromatin assembly factor 1 (CAF1) complex that delivers newly synthesized histone 3 (H3)/H4 dimers to host replication forks, is only partially understood (16, 17). EBV late genes are subsequently induced and include factors required for virion assembly and spread (10). Retrograde signals support ongoing lytic replication through subversion of chromatin-based repressors (18).

Most eBL cells utilize the latency I program, likewise enabling evasion of adaptive anti-EBV responses (19). Indeed, EBV was revealed as the first human tumor virus through eBL etiological studies, where the initial report noted that nearly all tumor cells did not produce infectious viral particles (20). With each S phase, EBV genomes are copied once by host cell machinery and are then partitioned to daughter cells (21). Histone octamers consisting of two copies of histone 2A (H2A), H2B, H3, and H4 are loaded onto leading and lagging strands. CAF1 has key roles in loading histones onto newly replicated and damaged host DNA, whereas the histone chaperone histone regulatory homologue A (HIRA) is important for nonreplicative histone loading onto host genomic sites (16, 17). Likewise, the chaperones alpha thalassemia/mental retardation syndrome X-linked chromatin remodeler (ATRX) and death domain-associated protein (DAXX) load histones onto telomeric and repetitive DNAs (22). EBV tegument protein BNRF1 downmodulates ATRX/DAXX activity in newly infected cells (23), but ATRX and DAXX subsequently acquire important roles in the suppression of EBV lytic reactivation in latently infected cells (24).

Here, we characterize histone chaperone roles of CAF1, HIRA, ATRX, and DAXX in Burkitt EBV latency. We provide evidence that type I and II EBV strains coopt each of these histone loaders to maintain latency via nonredundant roles. EBV upregulated each of the three CAF1 subunits in newly infected primary human B-cells, and CAF1 was found to have key roles in establishment of latency in a Burkitt EBV infection model. Chromatin immunoprecipitation (ChIP) assays support the notion of key CAF1 roles in deposition of repressive histone marks on EBV genome lytic control elements. These data further support the notion of key chromatin roles in regulation of the EBV lytic switch.

## RESULTS

### The histone loader CAF1 is important for Burkitt lymphoma EBV latency maintenance.

**FIG 1:**
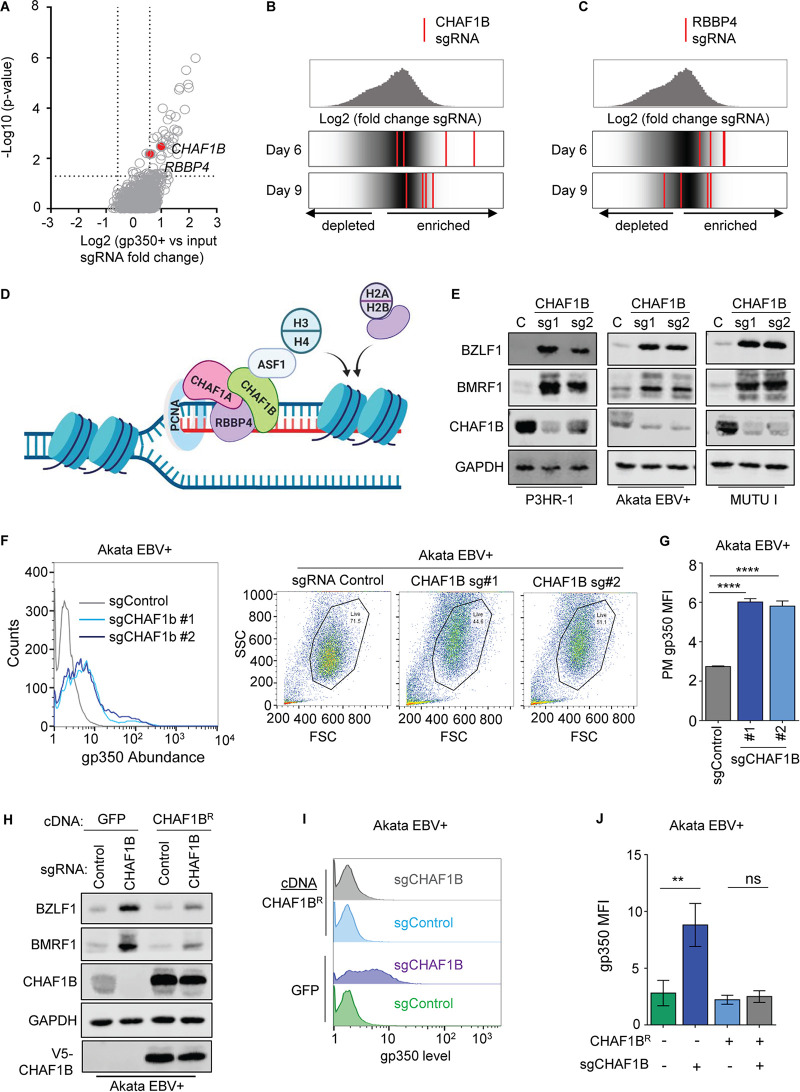
CHAF1B depletion triggers EBV lytic gene expression in Burkitt cells. (A) Volcano plot of CRISPR screen −Log_10_ (*P* value) and Log_2_ (fold change of gp350^+^ versus input library sgRNA abundance) values on day 6 after Avana library transduction (25). CAF1 subunits are indicated. (B and C) (Top) Distributions of Log_2_ (fold change gp350^+^ versus input library sgRNA abundance) values at day 6 (B) or day 9 (C) after sgRNA expression. (Bottom) Log_2_ fold change for the four CHAF1B-targeting (B) or RBBP4-targeting (C) sgRNAs (red lines), overlaid on gray gradients depicting overall sgRNA distributions at CRISPR screen day 6 versus day 9. Average values from two screen biological replicates are shown. (D) Model of DNA replication-dependent histone H3 and H4 loading by CAF1 and ASF1. Also shown are the CAF1 binding partner PCNA clamp and a histone chaperone loading histones H2A/H2B onto DNA. (E) Immunoblot analysis of whole-cell lysates (WCL) from P3HR-1, Akata EBV^+^, and MUTU I Burkitt cells expressing control or CHAF1B sgRNAs. See also Fig. S1B. (F) FACS analysis of plasma membrane (PM) gp350 expression and FSC/SSC plots in Akata cells expressing control or CHAF1B sgRNAs. Also shown to the right are FACS forward scatter (FSC) and side scatter (SSC) scatterplots, showing the live-cell gate used for measurement of gp350 abundances. (G) Mean + standard deviation (SD) PM gp350 mean fluorescence intensities (MFI) from *n* = 3 replicates determined as described for panel F. ****, *P* < 0.0001. (H) Immunoblot analysis of WCL from Akata EBV^+^ cells expressing GFP or V5 epitope-tagged CHAF1B rescue cDNA (CHAF1B^R^) and the indicated sgRNAs. (I) FACS analysis of PM gp350 expression in Akata EBV^+^ cells that stably express GFP or CHAF1B^R^ and the indicated sgRNAs. See also Fig. S1F for FACS FSC/SSC live-cell gates used. (J) Mean ± SD PM gp350 MFI values from *n* = 3 replicates determined as described for panel I. **, *P* < 0.01; ns, not significant. Blots in panels E and H are representative of *n* = 3 replicates.

To gain insights into host factors important for the maintenance of EBV latency, we recently performed a human genome wide CRISPR screen (25). Briefly, Cas9-positive (Cas9^+^) EBV^+^ Burkitt P3HR-1 cells were transduced with the Avana single guide RNA (sgRNA) library, which contains four independent sgRNAs against nearly all human genes. Cells with derepressed plasma membrane (PM) expression of the EBV late lytic antigen gp350, indicative of latency reversal, were sorted at days 6 and 9 postransduction. sgRNAs significantly enriched in the sorted population versus the input cell population were identified. The STARS algorithm identified 85 statistically significant hits at a *P* cutoff value of <0.05 and a fold change cutoff value of >1.5 (Fig. 1A) (25, 26). Unexpectedly, genes encoding two subunits of the histone loader CAF1 complex were among top screen hits (Fig. 1A to C).

10.1128/mBio.01063-20.1 FIG S1 CHAF1B depletion triggers expression of EBV lytic antigens in Burkitt cells. (A) (Top) Distribution of Log2 fold change (LFC) values of sgRNAs in gp350^+^ sorted versus input library cells for all Avana library guides at screen day 6. (Bottom) LFC values determined for the four *CHAF1A*-targeting sgRNAs (red lines), overlaid on a gray gradient depicting the overall sgRNA distribution, at CRISPR screen day 6 versus day 9. Average values from two screen biological replicates are shown. (B) Mean + standard deviation (SD) fold change of BZLF1/GAPDH intensity relative to sgControl cell levels, quantified from immunoblots from *n* = 3 experiments, including the experiment represented in Fig. 1E. (C) qRT-PCR analysis of selected viral immediate early, early, and late genes in Akata EBV^+^ cells expressing control or independent CHAF1B sgRNA (mean + SD values from *n* = 3 replicates; ****, *P* < 0.0001). (D) FACS analysis of PM gp350 expression in MUTU I cells expressing control or CHAF1B sgRNAs. (E) Mean + SD PM gp350 mean fluorescence intensity (MFI) values from *n* = 3 replicates of MUTU I with indicated sgRNAs determined as described for panel D. ****, *P* < 0.0001. (F) FACS FSC/SSC plots from Akata EBV^+^ with indicated GFP control or CHAF1B rescue (CHAF1B^R^) cDNAs and sgRNAs from the experiment represented in Fig. 1I. (G) Immunoblot analysis of WCL from MUTU I BL with the indicated cDNA and sgRNAs. Data are representative of results from *n* = 3 experiments. Download FIG S1, TIF file, 1.0 MB. Copyright © 2020 Zhang et al. 2020 Zhang et al. This content is distributed under the terms of the Creative Commons Attribution 4.0 International license.

The heterotrimeric CAF1 complex, comprised of CHAF1A, CHAF1B, and RBBP4 subunits, delivers histone H3/H4 dimers to the replication fork during the cell cycle S phase, typically together with histone chaperone ASF1a (17, 27) (Fig. 1D). CAF1 has well-established roles in maintenance of heterochromatin and cell identity, but its function in regulating EBV latency has not yet been investigated. Therefore, it was notable that multiple sgRNAs targeting *CHAF1B* and *RBBP4* were enriched among gp350^+^ sorted cells at days 6 and 9 post-Avana library transduction (Fig. 1B and C). A sgRNA targeting *CHAF1A* was also enriched in gp350^+^ cells at the day 6 time point (see Fig. S1A in the supplemental material). The identification of multiple sgRNAs targeting CAF1 subunits suggested an important CAF1 role in maintenance of EBV latency. Notably, Burkitt lymphomas are the fastest-growing human tumors, and newly synthesized EBV genomes must be reprogrammed to maintain the latency I program with each cell cycle.

To validate screen hits corresponding to CAF1 roles in the maintenance of BL EBV latency, control or independent *CHAF1B*-targeting sgRNAs were expressed in P3HR-1, Akata, and MUTU I Cas9^+^ tumor-derived endemic Burkitt lymphoma cell lines. In each of these, CHAF1B depletion induced immediate early BZLF1 and early BMRF1 expression (Fig. 1E; see also Fig. S1B). CHAF1B depletion significantly induced all seven EBV lytic transcripts measured by quantitative real-time PCR (qRT-PCR) (Fig. S1C). Since Akata and MUTU I harbor type I EBV, whereas P3HR-1 carries type II EBV, these data suggest conserved CAF1 roles in maintenance of EBV latency. Likewise, CHAF1B depletion induced gp350 plasma membrane expression on most Akata cells examined by flow cytometry (Fig. 1F and G), suggesting that Burkitt cell CAF1 loss triggers a full EBV lytic cycle. CHAF1B depletion also induced gp350 expression on MUTU I cells (Fig. S1D and E).

We next validated on-target CRISPR effects through a cDNA rescue approach. A point mutation was engineered into the *CHAF1B* cDNA protospacer-adjacent motif (PAM) site targeted by CHAF1B sgRNA 1 to abrogate Cas9 editing. EBV^+^ Akata cells with stable control green fluorescent protein (GFP) versus V5 epitope-tagged CHAF1B rescue cDNA (CHAF1B^R^) were established. Effects of control versus *CHAF1B*-targeting sgRNA were tested. Interestingly, depletion of endogenous CHAF1B derepressed BZLF1, BMRF1, and gp350 in control cells but failed to do so in cells with CHAF1B^R^ rescue cDNA expression (Fig. 1H to J; see also Fig. S1F). Similar cDNA rescue results for BZLF1 and BMRF1 expression were evident in MUTU I cells (Fig. S1G). These results suggest that CHAF1B is necessary for EBV latency in Burkitt cells, perhaps in loading histone H3/H4 onto newly synthesized episomes.

### CHAF1B perturbation induces EBV genome lytic replication and IFN-stimulated genes.

**FIG 2:**
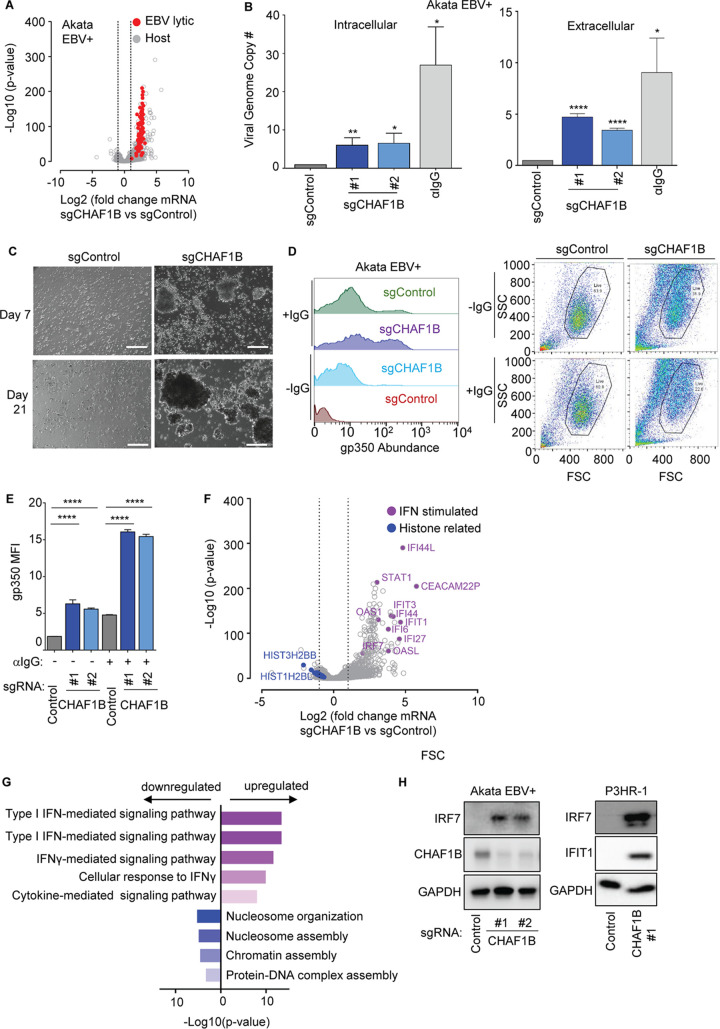
CHAF1B depletion triggers Burkitt cell EBV lytic reactivation and interferon-stimulated gene expression. (A) Volcano plot comparing RNAseq −Log_10_ (*P* value) versus Log_2_ (fold change sgCHAF1B versus sgControl mRNA abundance) from *n* = 3 replicates. Significantly changed EBV lytic gene values are shown in red; host genes are shown in gray. (B) qPCR analysis of EBV intracellular or DNase-treated extracellular genome copy number from Akata EBV^+^ cells expressing control or CHAF1B sgRNAs. Total genomic DNA was extracted at day 6 after lentivirus transduction or 48 h after stimulation by anti-IgG (10 μg/ml). Mean ± SD values from *n* = 3 biologically independent replicates are shown. *, *P* < 0.05; **, *P* < 0.01; ****, *P* < 0.0001. (C) Phase microscopy images of human primary B-cells at day 7 or 21 postinoculation with cell culture supernatant from Akata EBV^+^ cells expressing control or CHAF1B sgRNAs. White scale bar = 100 μm. (D) Representative FACS plots of PM gp350 expression and FSC/SSC in Akata EBV^+^ cells expressing control or CHAF1B sgRNAs in the absence or presence of anti-IgG (10 μg/ml) for 48 h. Also shown to the right are FACS FSC and SSC scatterplots, showing the live-cell gate used for measurement of gp350 abundances. (E) Mean ± SD PM gp350 MFI values from *n* = 3 replicates of Akata cells with indicated sgRNAs and anti-IgG stimulation determined as described for panel D. ****, *P* < 0.0001. (F) Volcano plot comparing RNAseq −Log_10_ (*P* value) versus Log_2_ (fold change sgCHAF1B versus sgControl mRNA abundance) values from *n* = 3 replicates. Purple circles indicate selected interferon (IFN)-stimulated genes, and blue circles indicate histone-related genes. (G) Enrichr pathway analysis of gene sets significantly upregulated (purple bars) or downregulated (blue bars) by CHAF1B sgRNA expression. Shown are the −Log_10_ (*P* values) data from Enrich analysis of triplicate RNAseq data sets, using Fisher’s exact test. See also Table S1. (H) Immunoblot analysis of WCL from Akata EBV^+^ or P3HR-1 cells expressing control or CHAF1B sgRNAs, for the IFN-stimulated genes IFIT1 and IRF7, CHAF1B, or GAPDH, as indicated. Blots shown in panel H are representative of *n* = 2 replicates.

EBV lytic replication is controlled on many levels, and partial lytic cycle induction is often observed. Therefore, we next examined whether CAF1 perturbation was sufficient to induce a productive lytic replication cycle. RNA sequencing (RNAseq) was performed on Akata EBV^+^ cells at day 6 post-sgRNA expression (see Table S1 in the supplemental material). RNAseq demonstrated significant induction of EBV 77 lytic cycle genes (Fig. 2A; see also Table S1). Consistent with induction of a full lytic cycle, CHAF1B depletion induced intracellular EBV genome amplification, albeit to a level less than that observed with Akata immunoglobulin (Ig) cross-linking. Likewise, CHAF1B sgRNAs induced secretion of DNase-resistant EBV genomes, demonstrating encapsidation (Fig. 2B). Similar results were observed in MUTU I and P3HR-1 cells, suggesting conserved CAF1 roles in type I and II EBV latency regulation (Fig. S2A and B). Furthermore, addition of supernatant from CHAF1B-depleted Akata cells but not from control Akata cells stimulated primary human B-cell aggregation and growth transformation (Fig. 2C). In support of the notion of on-target CRISPR effects, expression of the PAM site mutant CHAF1B cDNA rescue construct prevented EBV genome copy number increases with editing of endogenous *CHAF1B* (Fig. S2C) (28). To test whether CAF1 perturbation and Ig cross-linking synergistically induce lytic replication, control versus CHAF1B sgRNAs were expressed in Cas9^+^ Akata cells in the absence or presence of anti-IgG. Interestingly, Ig cross-linking induced higher levels of PM gp350 on live cells and increased intracellular/extracellular EBV genome copy numbers in cells depleted for CHAF1B compared to control cells (Fig. 2D and E; see also Fig. S2D). Similar results were obtained with IgM cross-linking in MUTU I cells (Fig. S2E to G). We note that Ig cross-linking induced a greater percentage of gp350-high cells (gp350 > 10^2^ [Fig. 2D]) than CHAF1B depletion alone, perhaps because B-cell receptor stimulation more robustly induced the lytic cycle, consistent with the viral genome copy number results shown in Fig. 2B. These results suggest that CAF1 not only maintains EBV latency in unstimulated cells but also limits the extent of lytic reactivation upon B-cell receptor activation.

10.1128/mBio.01063-20.2 FIG S2 Depletion of CHAF1B induces lytic reactivation in multiple EBV^+^ Burkitt tumor cell lines. (A to C) qPCR analysis of EBV intracellular or DNase-treated extracellular genome copy number from MUTU I (A), P3HR-1 (B), or Akata EBV^+^ (C) cells expressing control or CHAF1B sgRNAs. Total genomic DNA was extracted at day 6 after lentivirus transduction. Mean ± SD values from *n* = 3 replicates are shown. ****, *P* < 0.0001. (D) qPCR analysis of EBV intracellular or DNase-treated extracellular genome copy number from Akata EBV^+^ cells expressing GFP of V5-CHAF1B^R^ cDNAs and the indicated sgRNAs. Cells expressed GFP where not indicated to express CHAF1B^R^ and expressed sgControl where not indicated to express sgCHAF1B. Total genomic DNA was extracted at day 6 after lentivirus transduction. Mean + SD values from *n* = 3 replicates are shown. ****, *P* < 0.0001; *, *P* < 0.05; ns, nonsignificant. (D) qPCR analysis of EBV intracellular or extracellular genome copy number from Akata cells expressing control or CHAF1B sgRNAs compared alone or in combination with 10 μg/ml of anti-IgG for 48 h. Mean ± SD values from *n* = 3 replicates are shown. ****, *P* < 0.0001. (E) FACS analysis of PM gp350 expression in MUTU I cells expressing control or CHAF1B sgRNAs that were either mock-induced or induced with anti-IgM 10 μg/ml. (F) FACS FSC/SSC plots from MUTU1 cells expressing control or CHAF1B sgRNAs in the absence or presence of anti-IgG (10 μg/ml) for 48 h as described for panel E. Data are representative of results from *n* = 3 experiments. (G) qPCR analysis of EBV intracellular or DNase-treated extracellular genome copy number from MUTU I expressing control or CHAF1B sgRNAs alone or in combination with anti-IgM induction. KO cells were mock-treated or treated with 10 μg/ml of anti-IgM for 48 h. Mean + SD values from *n* = 3 replicates are shown. ****, *P* < 0.0001. Download FIG S2, TIF file, 0.9 MB. Copyright © 2020 Zhang et al. 2020 Zhang et al. This content is distributed under the terms of the Creative Commons Attribution 4.0 International license.

10.1128/mBio.01063-20.9 TABLE S1 Differentially expressed genes in EBV^+^ Akata cells expressing control or CHAF1B sgRNAs and Enrichr analysis of selected genes (adjusted *P* values of <0.05 and LFC values of more than 1 or less than −1). Download Table S1, XLSX file, 0.3 MB. Copyright © 2020 Zhang et al. 2020 Zhang et al. This content is distributed under the terms of the Creative Commons Attribution 4.0 International license.

RNAseq analysis also demonstrated robust upregulation of EBV latency III transcripts in response to CHAF1B depletion. EBNA1, EBNA2, EBNA3A, EBNA3B, EBNA3C, LMP1, LMP2A, and LMP2B were each significantly upregulated (Fig. S3A; see also Table S1). While these transcripts are upregulated by EBV lytic reactivation (28), the magnitude of mRNA upregulation suggests that CAF1 may also have important roles in chromatin-based silencing of the latency III program. We next examined changes in host mRNAs following CHAF1B depletion. Interestingly, multiple interferon (IFN)-stimulated genes (ISGs), including the IFIT1, IFIT3, IFI44, IFI44L, IRF7, and STAT1 genes, were among the most highly CHAF1B sgRNA-induced host genes, and GO analysis identified the type I interferon-mediated signaling pathway as the most highly upregulated pathway (Fig. 2F and G; see also Fig. S3B) (Table S1). CHAF1B-mediated upregulation of IRF7 and IFIT1 was validated at the protein level (Fig. 2H). Interestingly, ISG upregulation was not observed at the mRNA level or protein level in Akata cells upon immunoglobulin cross-linking-induced EBV lytic reactivation (29–31). These results suggest that EBV lytic replication itself does not underlie this host response, at least when triggered by Ig cross-linking.

10.1128/mBio.01063-20.3 FIG S3 RNAseq heat map analyses of CHAF1 depletion effects on Akata EBV latency III gene, interferon-stimulated gene, and histone gene expression. (A) Heat map representation of EBV latency III gene abundance in Akata EBV^+^ cells expressing control or CHAF1B sgRNAs. Shown are data from *n* = 3 biologically independent replicates. (B) Heat map representation of interferon-stimulated genes whose expression was significantly upregulated in sgCHAF1b-expressing versus sgControl-expressing Akata EBV^+^ cells. Shown are data from *n* = 3 replicates. (C) Heat map representation of mRNAs encoding histones whose expression was significantly different in Akata EBV^+^ cells expressing sgCHAF1B versus sgControl. Shown are data from *n* = 3 replicates. Histone H3 genes are labeled in red. (D) Immunoblot of whole-cell extracts from Akata EBV^+^ cells expressing the indicated control, CHAF1B, or MYC sgRNAs. Data are representative of results from *n* = 3 replicates. Download FIG S3, TIF file, 1.3 MB. Copyright © 2020 Zhang et al. 2020 Zhang et al. This content is distributed under the terms of the Creative Commons Attribution 4.0 International license.

Consistent with CRISPR effects at the level of the CAF1 histone chaperone complex, RNAseq gene ontology analysis showed that, perhaps as a result of a negative feedback in response to diminished CAF1 activity, the only host pathways downregulated by CHAF1B sgRNA at an adjusted *P* cutoff value of <0.05 were nucleosome assembly (GO:0006334), chromatin assembly (GO:0031497), and nucleosome organization (GO:0034728) (Fig. 2F and G; see also Fig. S3C). While we recently reported that depletion of the histone chaperone facilitated chromatin transcription (FACT) induces lytic reactivation by impairing MYC expression (25), we observed only modest reduction of MYC RNA or protein with CHAF1B depletion (Table S1; see also Fig. S3D).

### Depletion of CAF1 subunits CHAF1A and RBBP4 triggers EBV lytic replication.

CHAF1B assembles together with CHAF1A and RBBP4 subunits with 1:1:1 stoichiometry (32). CHAF1A targets CAF1 to the replication fork through interaction with proliferating cell nuclear antigen (PCNA), associates with histone deacetylases, and has roles in DNA repair and in heterochromatin maintenance (17, 33). While RBBP4 has been implicated in CAF1 activity (34), it also has additional epigenetic roles, including within the NURD transcriptional repressor complex (35).

**FIG 3:**
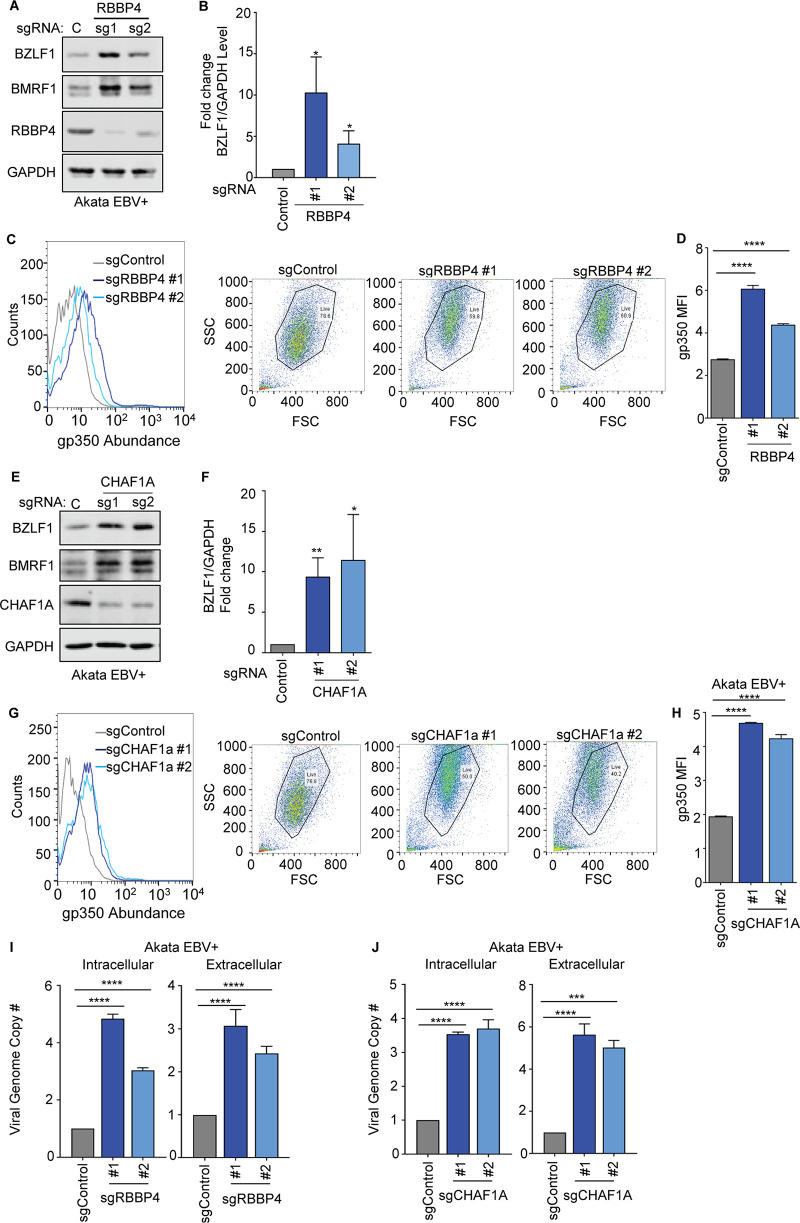
CAF1 subunits RBBP4 and CHAF1A are necessary for Burkitt cell EBV latency. (A) Immunoblot analysis of WCL from Akata EBV^+^ cells expressing control or RBBP4 sgRNAs. (B) Mean + SD fold change of BZLF1/GAPDH intensity relative to sgControl cell levels, quantified from immunoblots from *n* = 3 experiments, including that represented in panel A. (C) FACS analysis of plasma membrane (PM) gp350 expression and FSC/SSC plots in Akata EBV^+^ cells expressing control or RBBP4 sgRNAs. Also shown to the right are FACS scatterplots, showing the live-cell gate used for measurement of gp350 abundances. (D) Mean + SD PM gp350 MFI values from *n* = 3 replicates of Akata EBV^+^ with the indicated sgRNAs determined as described for panel C. ****, *P* < 0.0001. (E) Immunoblot analysis of WCL from Akata cells expressing control or CHAF1A sgRNAs. (F) Mean + SD fold change of BZLF1/GAPDH intensity relative to sgControl cell levels, quantified from immunoblots from *n* = 3 experiments, including that represented in panel B. (G) FACS analysis of plasma membrane (PM) gp350 expression and FSC/SSC plots in Akata EBV^+^ cells expressing control or CHAF1a sgRNAs. Also shown to the right are FACS scatterplots, showing the live-cell gate used for measurement of gp350 abundances. (H) Mean + SD PM gp350 MFI values from *n* = 3 replicates of Akata EBV^+^ cells with the indicated sgRNAs determined as described for panel G. ****, *P* < 0.0001. (I and J) qPCR analysis of EBV intracellular or DNase-treated extracellular genome copy number from Akata EBV^+^ cells expressing control, RBBP4 (I), or CHAF1A (J) sgRNAs. Total genomic DNA was extracted at day 6 after lentivirus transduction. Mean ± SD values from *n* = 3 replicates are shown. ****, *P* < 0.0001.

To investigate whether CAF1 subunits CHAF1A and RBBP4 were similarly important for the maintenance of the Burkitt EBV latency I program, we tested the effects of the top two Avana library sgRNAs targeting the genes encoding each. Depletion of RBBP4 or CHAF1A by either sgRNA induced all seven EBV lytic genes surveyed by quantitative PCR (qPCR) (Fig. S4A and B) and induced BZLF1, BMRF1, and gp350 at the protein level (Fig. 3A to H). RBBP4 or CHAF1A depletion likewise derepressed EBV lytic gene expression in MUTU 1 and P3HR-I (Fig. S4C and D). To determine effects of RBBP4 or CHAF1A depletion on EBV genome amplification, viral load analysis was performed. RBBP4 and CHAF1A sgRNAs significantly increased intracellular and DNase-treated extracellular EBV genome copy numbers in three Burkitt cell lines (Fig. 3I and J; see also Fig. S4E and F). Taken together, these results suggest that all three CAF1 subunits are critical for EBV latency in Burkitt lymphoma cells, perhaps acting to reprogram newly synthesized EBV episomes with each cell cycle.

10.1128/mBio.01063-20.4 FIG S4 CHAF1A or RBBP4 depletion triggers Burkitt cell EBV lytic reactivation. (A and B) qRT-PCR analysis of selected viral immediate early, early, and late genes from Akata EBV^+^ cells expressing control or independent RBBP4 (A) or CHAF1A (B) sgRNAs. Mean ± SD values from *n* = 3 replicates are shown. ****, *P* < 0.0001. (C) Immunoblot analysis of WCL from MUTU I cells expressing control or independent CHAF1A sgRNAs. Blots are representative of *n* = 3 replicates. Shown at right are data representing mean + SD fold change of BZLF1/GAPDH intensity relative to sgControl cell levels, quantified from immunoblots from *n* = 3 experiments, including the ones shown on the left. (D) Immunoblot analysis of WCL from MUTU I cells expressing control or independent RBBP4 sgRNAs. Blots are representative of *n* = 3 replicates. Shown at right are data representing mean + SD fold change of BZLF1/GAPDH intensity relative to sgControl cell levels, quantified from immunoblots from *n* = 3 experiments, including the ones shown on the left. (E) qPCR analysis of EBV intracellular or DNase-treated extracellular genome copy number from MUTU I cells expressing control, CHAF1A, or RBBP4 sgRNAs. Mean +SD values from *n* = 3 replicates are shown. ****, *P* < 0.0001; ***, *P* < 0.001. (F) qPCR analysis of EBV intracellular or DNase-treated extracellular genome copy number from P3HR-1 cells expressing control, CHAF1A, or RBBP4 sgRNAs. Mean + SD values from *n* = 3 replicates are shown. ****, *P* < 0.0001; ***, *P* < 0.001. Download FIG S4, TIF file, 0.8 MB. Copyright © 2020 Zhang et al. 2020 Zhang et al. This content is distributed under the terms of the Creative Commons Attribution 4.0 International license.

### EBV-induced CAF1 expression.

**FIG 4:**
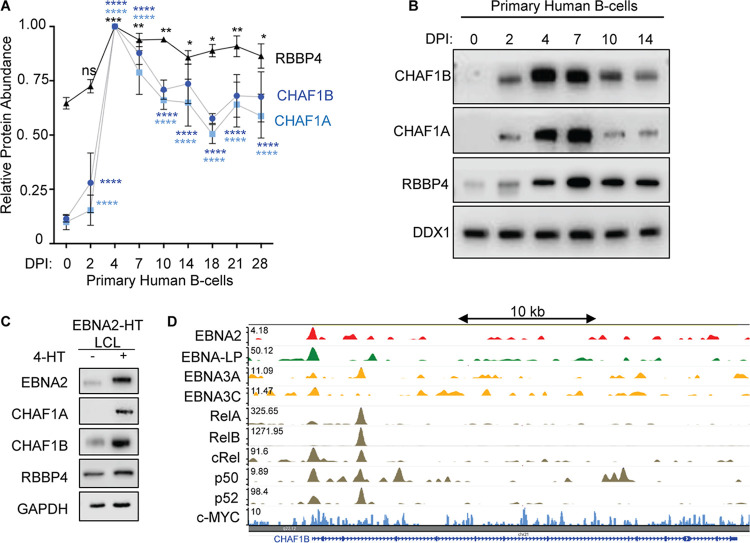
CAF1 complex restricts lytic cycle after EBV infection in primary human B-cells. (A) CHAF1B, CHAF1A, and RBBP4 relative protein abundances detected by tandem-mass-tag-based proteomic analysis of primary human B-cells at rest and at nine time points after EBV B95.8 infection at a multiplicity of infection of 0.1. Data represent averages ± standard errors of the means (SEM) of results from *n* = 3 independent replicates (36). For each protein, the maximum level detected across the time course was set to a value of 1. (B) Immunoblot analysis of WCL from primary B-cells infected with B95.8 EBV at days 0, 2, 4, 7, 10, and 14 postinfection. Data are representative of results from *n* = 3 experiments. (C) Immunoblot analysis of WCL from 2-2-3 EBNA2-HT LCLs with conditional EBNA2 expression, cultured in the absence or presence of 4-hydroxytamoxifen (4-HT; 1 μM) for 48 h. Data are representative of results from *n* = 3 experiments. (D) GM12878 ChIP-seq signals of EBV-encoded EBNA2, EBNA-LP, EBNA3A, EBNA3C, LMP1-activated RelA, RelB, cRel, p50, p52 NF-κB subunits or c-Myc at the *CHAF1B* locus. Track heights are indicated at the upper left.

Since CAF1 has key histone deposition roles in the context of DNA replication or repair, we asked whether CAF1 subunits are expressed in resting or in newly infected primary human B-cells. Using data from recently published RNAseq and proteomic maps of EBV-mediated primary B-cell growth transformation (36, 37), we noticed that there was little expression of CHAF1A or CHAF1B in resting B-cells but that each was strongly upregulated by 2 days postinfection (dpi) (Fig. 4A; see also Fig. S5A). At that early postinfection time point, EBNA2 and EBNA-LP are the major EBV proteins expressed and there is little LMP1 expression or dependence on NF-κB activation (36–39). RBBP4 appeared to have a higher basal level, perhaps reflective of its additional epigenetic roles beyond CAF1, but was also EBV upregulated (Fig. S5A). Immunoblot analysis demonstrated strong CHAF1B upregulation between 2 and 4 days postinfection (Fig. 4B), a time period which EBNA2 drives newly infected cells to rapidly proliferate as they transition from the EBV prelatency program to latency IIb (12, 40).

10.1128/mBio.01063-20.5 FIG S5 CAF1 mRNA levels in newly EBV-infected B-cells and ChIP-seq signals in LCLs. (A) Normalized CHAF1B, CHAF1A, or RBBP4 mRNA levels from primary human peripheral blood B-cells at the indicated day postinfection (dpi) by the EBV B95.8 strain (37). Shown are the means + standard errors of the means (SEM) of results from *n* = 3 biologically independent RNAseq datasets. ****, *P* < 0.0001; ***, *P* < 0.001; **, *P* < 0.01. (B and C) LCL ChIP-seq signals of EBNA2, EBNALP, EBNA3A, EBNA3C, RelA, RelB, cRel, p50, p52, and MYC at the *CHAF1A* (B) or *RBBP4* (C) loci. Track heights are indicated in the upper left, and genomic positions are indicated at top of each panel. Download FIG S5, TIF file, 0.8 MB. Copyright © 2020 Zhang et al. 2020 Zhang et al. This content is distributed under the terms of the Creative Commons Attribution 4.0 International license.

We hypothesized that EBNA2 may be a major viral inducer of CAF1 subunit expression in EBV-infected B-cells, given the kinetics of their upregulation. To test this hypothesis, we made use of 2-2-3 EBNA2-HT lymphoblastoid cell lines (LCLs) (here referred to as EBNA2-HT). In this cell line, 4-hydroxytamoxifen (4-HT) positively regulates the nuclear localization and stability of a conditional EBNA2 allele, where EBNA2 is fused to a mutant estrogen-receptor-ligand-binding domain (41, 42). Conditional inactivation of EBNA2-HT by 4-hydroxytamoxifen (4HT) withdrawal strongly diminished expression of CHAF1A, CHAF1B, and, to a lesser extent, RBBP4 protein (Fig. 4C). Consistent with this result, conditional EBNA2 inactivation reduces CAF1 subunit expression at the mRNA level (43). Published LCL chromatin immunoprecipitation sequencing (ChIP-seq) data (44–47) show that EBNA-LP, EBNA3A, EBNA3C, and LMP1-activated NF-κB subunits cooccupy *CHAF1A*, *CHAF1B*, and *RBBP4* promoters (Fig. 4D; see also Fig. S5B and C), suggesting that they may also support CAF1 expression. Likewise, MYC cooccupies the *CHAF1A* and *RBBP4* promoters in Burkitt-like P493 B-cells (48) (Fig. S5B and C).

### The histone chaperone HIRA exerts nonredundant Burkitt cell maintenance of EBV latency roles.

The histone loader histone regulatory homologue A (HIRA) interacts with ASF1a and preferentially loads histone H3.3/H4 complexes onto DNA in a replication-independent manner throughout the cell cycle, for example, at areas of active transcription (17, 49). HIRA regulates latency of the alphaherpesvirus herpes simplex virus and the betaherpesvirus cytomegalovirus (50–53). HIRA is also implicated in maintenance of HIV latency (54) but to our knowledge has not been investigated in the regulation of gammaherpesvirus latency.

**FIG 5:**
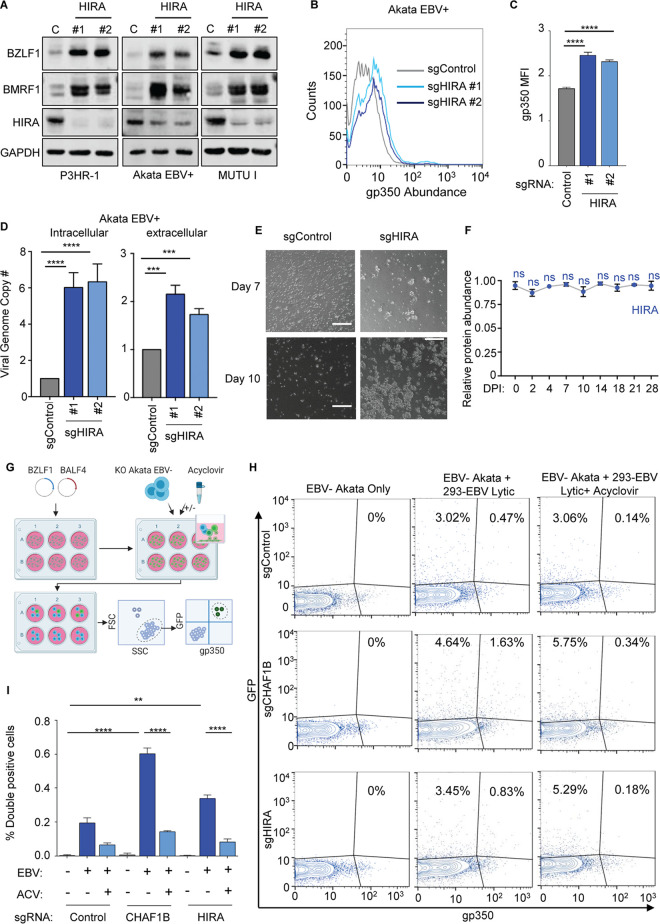
Histone 3.3 chaperone HIRA restricts Burkitt EBV lytic reactivation. (A) Immunoblot analysis of WCL from P3HR-1, Akata EBV^+^, or MUTU I BL cells expressing control or HIRA sgRNAs. (B) FACS analysis of PM gp350 expression in Akata EBV-positive cells expressing control or HIRA sgRNAs. (C) Mean + SD PM gp350 MFI values from *n* = 3 replicates of Akata cells with indicated sgRNAs determined as described for panel B. ****, *P* < 0.0001. (D) qPCR analysis of EBV intracellular or DNase-resistant extracellular genome copy number from Akata EBV^+^ cells expressing control or HIRA sgRNAs. Total genomic DNA was extracted at day 6 after lentivirus transduction. Mean + SD values from *n* = 3 replicates are shown. ****, *P* < 0.0001. (E) Phase microscopy images of human primary B-cells at day 7 or 10 postinoculation with cell culture supernatant from Akata cells expressing control or HIRA sgRNAs. White scale bar = 100 μm. (F) HIRA relative protein abundances detected by tandem-mass-tag-based proteomic analysis of primary human B-cells at rest and at nine time points after EBV B95.8 infection at a multiplicity of infection of 0.1. Data represent averages ± SEM for *n* = 3 independent replicates (36). For each protein, the maximum level detected across the time course was set to a value of 1. (G) Schematic diagram of cell coculture system for newly infected Burkitt cell EBV latency establishment. HEK-293 EBV^+^ 2-8-15 cells, which harbor a recombinant viral genome with GFP marker, were triggered for lytic reactivation by transfection with BZLF1 and BALF4 expression vectors. At 48 h later, the induced HEK-293 EBV^+^ cells were then cocultured with EBV-negative (EBV^−^) Akata cells, in the absence or presence of acyclovir (at that point, HEK-293 cells were already producing EBV, but acyclovir was able to block late gene expression in newly infected Akata cells). At 48 h later, cells are analyzed by FACS analysis for expression of GFP and the late lytic antigen gp350, which is expressed in newly infected cells that fail to establish latency in the absence of acyclovir. (H) Control, CHAF1B KO or HIRA KO Akata EBV^−^ cells were cocultured with HEK-293 2-8-15 cells that had been transfected 48 h previously with BZLF1 and BALF4 expression vectors. Cells were mock-treated or treated with 100 μg/ml of acyclovir at the time of coculture. Cells were then subjected to FACS analysis for GFP or PM gp350. GFP versus gp350 dot plots from a representative replicate are shown. (I) Mean + SD PM gp350 MFI values from *n* = 3 replicates of Akata EBV^−^ cells cocultured with HEK-293 EBV^+^ cells as described for panels G and H, under the indicated experimental conditions. ****, *P* < 0.0001. ACV = acyclovir.

To explore potential HIRA roles in the maintenance of EBV latency, we tested effects of HIRA depletion in P3HR-1, Akata, and MUTU I cells. In each of these BL cell lines, CRISPR *HIRA* editing by either of two Avana sgRNAs rapidly upregulated BZLF1 and BMRF1 in EBV^+^ Akata, P3HR-1, and MUTU I cells (Fig. 5A; see also Fig. S6A). HIRA depletion also upregulated PM gp350 abundance, albeit to a lesser extent than observed with CAF1 perturbation, perhaps explaining why our CRISPR screen was more sensitive to CAF1 perturbation (Fig. 5B and C). HIRA sgRNAs increased expression of all seven EBV lytic mRNAs quantified by qPCR (Fig. S6B) as well as intracellular and extracellular EBV genome copy number in EBV^+^ Akata cells (Fig. 5D). Supernatants from HIRA-depleted Akata cells induced primary human B-cell clumping, though the clusters were generally smaller than those observed with supernatants from equal numbers of CHAF1B-depleted cells, likely reflecting lower titer of secreted EBV (Fig. 5E). Taken together, these results indicated that HIRA and CHAF1B have nonredundant roles in the maintenance of Burkitt EBV latency, as depletion of either triggers lytic reactivation and production of infectious virus. In contrast to CHAF1A and CHAF1B, HIRA mRNA and protein abundance was not significantly changed by primary human B-cell EBV infection, perhaps suggesting that HIRA is well positioned to regulate incoming EBV genomes (Fig. 5F; see also Fig. S6C).

10.1128/mBio.01063-20.6 FIG S6 HIRA depletion triggers Burkitt cell EBV lytic reactivation. (A) Mean + SD fold change of BZLF1/GAPDH intensity, relative to sgControl cell levels, quantified from immunoblots from *n* = 3 experiments in Akata EBV^+^, MUTU1, and P3HR-1 cells expressing control sgRNAs, HIRA sgRNAs, RBBP4 sgRNAs, or CHAF1B sg RNAs, including those from the experiment represented in Fig. 5A. *, *P* < 0.05; **, *P* < 0.01; ****, *P* < 0.0001. (B) qRT-PCR analysis of selected viral immediate early, early, and late genes from Akata EBV^+^ cells expressing control or HIRA sgRNAs. Data represent means + SD of results from *n* = 3 replicates. ****, *P* < 0.0001. (C) Normalized HIRA mRNA levels in primary human peripheral blood B-cells at the indicated day postinfection (dpi) by EBV B95.8 (37). Shown are the mean + SD values from *n* = 3 of biologically independent RNAseq replicates. *, *P* < 0.05; ns, nonsignificant. (D) FACS scatterplots showing the live-cell gate used for measurement of gp350 abundances in EBV-negative Akata cells that were cocultured with HEK-293 EBV^+^ cells induced for lytic replication by transfection with BZLF1/BALF4 expression vectors for 48 h, followed by acyclovir treatment (100 μg/ml) as indicated as described for Fig. 5H. Data are representative of results from *n* = 3 experiments. (E) Immunoblot analysis of WCL from Akata EBV^−^ cells cocultured with HEK-293 cells under the indicated experimental conditions from the experiment represented in Fig. 5H. Data are representative of results from *n* = 3 experiments. Download FIG S6, TIF file, 1.2 MB. Copyright © 2020 Zhang et al. 2020 Zhang et al. This content is distributed under the terms of the Creative Commons Attribution 4.0 International license.

We next cross-compared putative CAF1 or HIRA roles in the establishment of EBV latency. Since it is not currently possibly to do CRISPR editing in resting primary B-cells, we instead used an EBV-negative (EBV^−^) subclone of Akata Burkitt cells, which were established during serial passage of the original EBV^+^ Akata tumor cells (55). It has previously been shown that latency I is established upon reinfection of these cells by EBV *in vitro* (56). However, since EBV^−^ Akata cells are difficult to infect with purified EBV, we developed a coculture system to increase infection efficiency (Fig. 5G). EBV^−^ Akata cells were cocultured with EBV^+^ HEK-293 producer cells, which carry a recombinant EBV bacterial artificial chromosome (BAC) system that includes a GFP marker (57). Lytic replication was induced in a monolayer of adherent HEK-293 EBV^+^ cells by transfection of genes encoding BZLF1 and BALF4. Induced HEK-293 cells were then cocultured with Akata EBV^−^ cells at 48 h posttransfection. EBV infection frequency was monitored by fluorescence-activated cell sorter (FACS) analysis 48 h later, using GFP as a readout, and PM gp350 positivity was used as a marker for Akata cells with lytic replication.

Using this coculture system, ∼3.5% of control Akata cells were infected, as judged by expression of the GFP marker, and 0.47% were positive for the gp350 lytic antigen. By comparison, ∼6% of CHAF1B-depleted cells were infected as judged by the GFP marker and ∼1.6% had gp350 PM expression (Fig. 5H and I; see also Fig. S6D). HIRA sgRNA expression increased the percentage of gp350^+^ cells among newly infected GFP^+^ Akata cells (Fig. 5H and I), albeit less robustly than CHAF1B sgRNA. We speculate that the higher rate of EBV infection in CHAF1B-depleted cells may have resulted from increased expression of the EBV coreceptor major histocompatibility complex (MHC) class II (Table S1), including a 4-fold increase in HLA-DP, a 3-fold increase in HLA-DRA, a 2-fold increase in HLA-DMA, a 1.7-fold increase in HLA-DPB1, and significant increases in transcripts of the MHC class II pathway regulators HLA-DM and HLA-DO.

Akata cell gp350 signal in this coculture system might result from adherence of EBV virion to the cell surface versus resulting from new gp350 expression from the late lytic *BLLF1* gene. To distinguish between these two possibilities, acyclovir was added at the coculture system, at the time at which previously induced HEK-293 EBV^+^ cells were added to Akata EBV^−^ cells, therefore allowing the HEK-293 cells to continue to secrete EBV, at least initially. Acyclovir significantly reduced gp350 expression in control and CHAF1B-depleted or HIRA-depleted cells (Fig. 5H and I). Yet, Akata cells continued to be infected by EBV, as judged by the acquisition of the GFP marker, whose expression is induced in newly infected cells. These results suggest that gp350 was expressed as a late lytic gene, rather than being delivered by incoming or attached EBV, in newly infected Akata cells (Fig. 5H and I). Further suggesting an important CAF1 role in establishment of latency in Akata cells, CHAF1B depletion induced BZLF1 expression in newly infected Akata cells (Fig. S6E). Thus, our results are consistent with a model in which HIRA and CAF1 have nonredundant roles in regulation of EBV latency in Burkitt cells.

The histone H3.3 loaders ATRX and DAXX have roles in telomeres and have been implicated in maintenance of Burkitt B-cell EBV latency. shRNA targeting of either ATRX or DAXX induces lytic antigen expression (24). Consistent with these RNA interference (RNAi) results, we found that CRISPR targeting of either ATRX or DAXX induced BZLF1 and BMRF1 expression on the mRNA and protein levels but more weakly induced plasma membrane gp350 expression (Fig. S7). Whereas sgRNAs targeting DAXX induced ∼2.5-fold increases in EBV copy number, ATRX-targeting sgRNAs failed to do so (Fig. S7). Collectively, these data indicate that multiple histone loaders have nonredundant roles in maintenance of EBV latency.

10.1128/mBio.01063-20.7 FIG S7 DAXX and ATRX depletion triggers Burkitt EBV lytic reactivation. (A) Immunoblot analysis of WCL from EBV^+^ Akata, MUTU I, or P3HR-1 cells expressing control or independent ATRX sgRNAs. (B) Immunoblot analysis of cell extracts from EBV^+^ Akata, MUTU I, or P3HR-1 cells expressing control or independent DAXX sgRNAs. (C) FACS analysis of PM gp350 expression in Akata EBV-positive cells expressing control, ATRX, or DAXX sgRNAs. (D) Mean + SD PM gp350 MFI values from *n* = 3 replicates of Akata EBV^+^ cells expressing control, ATRX, or DAXX sgRNAs determined as described for panel C. ****, *P* < 0.0001. (E and F) qRT-PCR analysis of selected viral immediate early, early, and late genes in Akata EBV^+^ cells expressing control or independent ATRX (E) or DAXX (F) sgRNAs. Data represent means + SD of results from *n* = 3 replicates; ****, *P* < 0.0001. (G) qPCR analysis of EBV intracellular genome copy number from Akata EBV^+^ cells expressing control, ATRX, or DAXX sgRNAs. Mean + SD values from *n* = 3 replicates are shown. ****, *P* < 0.0001; ns, nonsignificant. (H) ATRX or DAXX relative protein abundances detected by tandem-mass-tag-based proteomic analysis of primary human B-cells at rest and at nine time points after EBV B95.8 infection at a multiplicity of infection of 0.1. Data represent averages ± SEM for *n* = 3 independent replicates (36). For each protein, the maximum level detected across the time course was set to a value of 1. Blots in panels A and B are representative of results from *n* = 3 replicates. Download FIG S7, TIF file, 1.2 MB. Copyright © 2020 Zhang et al. 2020 Zhang et al. This content is distributed under the terms of the Creative Commons Attribution 4.0 International license.

### Loss of CHAF1B reduces the occupancy of H3.1 and H3.3 at promoters of EBV lytic genes.

CAF1 preferentially loads H3.1/H4 histone tetramers onto newly synthesized or damaged host DNA, though whether it is important for H3.1 loading onto latent EBV genomes remains unknown. In addition, little is presently known about whether histone H3.1 occupies key EBV genomic sites in latency compared to histone H3.3. We therefore used chromatin immunoprecipitation (ChIP) for analysis of endogenous histone 3.1 versus 3.3 and qPCR to investigate effects of CHAF1B depletion on their occupancy at key EBV genomic sites.

**FIG 6:**
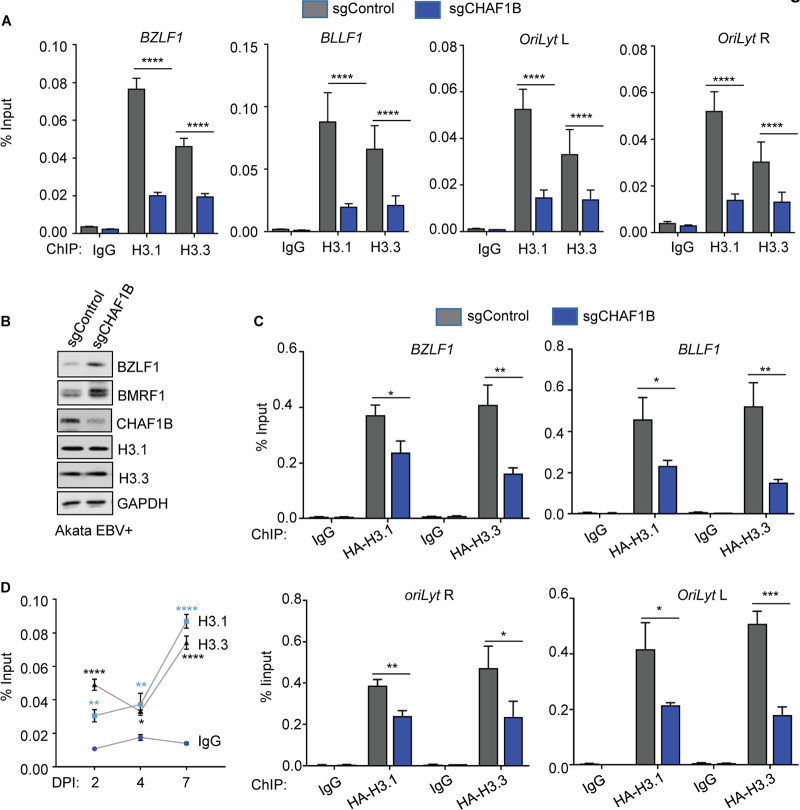
CHAF1B depletion reduces histone H3.1 and H3.3 occupancy at key EBV lytic cycle regulatory elements. (A) ChIP was performed using antibodies against endogenous H3.1 or H3.3 on chromatin from Akata EBV^+^ cells expressing control or CHAF1B sgRNA, followed by qPCR performed with primers specific for the *BZLF1* or *BLLF1* promoters, *oriLyt* R, or *oriLyt* L. Mean + SEM values are shown for *n* = 3 biologically independent replicates. *P* values were calculated by two-way ANOVA with Sidak’s multiple-comparison test. (B) Immunoblot analysis of WCL from EBV^+^ Akata BL cells expressing control or independent CHAF1B. (C) ChIP for HA epitope-tagged H3.1 or H3.3 using anti-HA antibody and chromatin from Akata EBV^+^ cells stably expressing HA-H3.1 or HA-H3.3 and the indicated sgRNAs. qPCR was then performed with primers specific for the *BZLF1* or *BLLF1* promoters, *oriLyt* R, or *oriLyt* L. Mean + SEM values are shown for *n* = 3 biologically independent replicates. *P* values were calculated by two-way ANOVA with Sidak’s multiple-comparison test. (D) ChIP for endogenous H3.1 or H3.3 was performed using antibodies targeting H3.1 or H3.3 on chromatin from human primary B-cells infected with B95.8 EBV at 2, 4, and 7 days postinfection, followed by qPCR performed with primers specific for the *BZLF1* promoter. Input DNA for each time point was normalized for intracellular EBV genome copy number. Mean ± SEM values are shown for *n* = 3 biologically independent replicates. *P* values were calculated by two-way ANOVA with Sidak’s multiple-comparison test.

CHAF1B depletion significantly decreased histone 3.1 (H3.1) occupancy at the immediate early *BZLF1* promoter and at the late gene *BLLF1* (encodes gp350) promoter. Likewise, sgCHAF1B expression decreased H3.1 occupancy at both origins of lytic replication (*oriLyt L* and *oriLyt R*), which are EBV genomic enhancers with key roles in lytic gene induction and in lytic DNA replication (58–60) (Fig. 6A). Similar results were obtained in acyclovir-treated cells, suggesting that production of unchromatinized lytic genomes did not falsely lower the effect represented by the ChIP-qPCR result (Fig. S8A). These data suggest that latent EBV genomes may be broadly occupied by H3.1-containing nucleosomes, most likely loaded in a DNA replication-dependent manner in S-phase (17, 61).

10.1128/mBio.01063-20.8 FIG S8 CHAF1B depletion reduces H3.1 loading at multiple EBV genomic lytic cycle regulatory elements in the presence of acyclovir. (A) ChIP for H3.1 or H3.3 was performed using antibodies targeting endogenous H3.1 or H3.3 on chromatin from Akata EBV^+^ cells expressing control or CHAF1B sgRNAs, treated with 100 μg/ml acyclovir. qPCR was performed with primers specific for *BZLF1* or *BLLF1* promoters, *oriLyt R*, or *oriLyt L*. Means ± SEM of results from *n* = 3 biologically independent replicates are shown. ****, *P* < 0.0001; **, *P* < 0.01. *P* values were calculated by two-way ANOVA with Sidak’s multiple-comparison test. (B) ChIP for UHRF1 was performed on chromatin from Akata EBV^+^ cells expressing control or CHAF1B sgRNAs, followed by qPCR performed with primers specific for EBV genomic promoter *Cp* or *Wp*. Means + SEM of results from *n* = 3 biologically independent replicates are shown. *P* values were calculated by two-way ANOVA with Sidak’s multiple-comparison test. (C) MeDIP was performed on chromatin from Akata EBV^+^ cells expressing control or CHAF1B sgRNAs treated with 100 μM acyclovir to prevent synthesis of unmethylated lytic EBV genomes, followed by qPCR performed with primers specific for the *BLLF1* promoters, *oriLyt R*, or *oriLyt L*. Means + SEM of results from *n* = 3 biologically independent replicates are shown. *P* values were calculated by two-way ANOVA with Sidak’s multiple-comparison test. Download FIG S8, TIF file, 0.3 MB. Copyright © 2020 Zhang et al. 2020 Zhang et al. This content is distributed under the terms of the Creative Commons Attribution 4.0 International license.

CHAF1B depletion also reduced H3.3 levels at *BZLF1*, *BLLF1*, and *oriLyt* sites, suggesting that CAF1 directly or indirectly also controls H3.3 loading onto latent EBV genomes. With regard to the latter possibility, RNAseq analysis demonstrated that sgCHAF1B expression diminished the expression of histone and histone-like genes, as well as that of the ATRX transcript by ∼30%, but modestly increased DAXX and HIRA mRNA levels (Table S1). We also note that upon lytic induction caused by CHAF1B depletion, the EBV early gene encoding BNRF1 is expressed, which can then disrupt ATRX/DAXX complexes to diminish H3.3 loading at these EBV genomic sites (23, 24, 62). Although CHAF1B depletion diminished expression of multiple histone and histone-related genes (Fig. 2F; see also Fig. S3C) (Table S1), sgCHAF1B expression did not reduce the steady-state H3.1 level or H3.3 level in EBV^+^ Akata cells, as judged by immunoblot analysis (Fig. 6B).

To next enable additional cross-comparison of CHAF1B perturbation effects on EBV genomic H3.1 and H3.3 occupancy using a single monoclonal antibody and to validate endogenous histone H3 ChIP results, we established EBV^+^ Akata cells with stable hemagglutinin (HA) epitope-tagged H3.1 or H3.3 expression (52). ChIP was then performed using monoclonal anti-HA antibody in cells expressing a single guide control (sgControl) versus sgCHAF1B. Consistent with observations using antibodies against endogenous H3.1 and H3.3, CHAF1B depletion similarly reduced HA-3.1 and HA-3.3 signals at *BZLF1*, *BLLF1*, and *oriLyt* sites (Fig. 6C). These results further suggest that the EBV genome is occupied by nucleosomes containing H3.1 and H3.3 in latency I.

To gain insights into histone H3 isoform loading onto EBV genomes in newly infected primary human B-cells, ChIP-qPCR analyses were performed at 2, 4, and 7 days after EBV infection. CD19^+^ B-cells were purified by negative selection and infected with B95.8 EBV at a multiplicity of infection (MOI) of 0.2.

By day 2, at which time point infected cells have undergone remodeling but have not yet divided and most cells should contain only 1 or 2 EBV genomes (63), H3.1 and H3.3 loading levels were already significantly increased. This result suggests that both H3 isoforms are loaded onto incoming EBV genomes, potentially by multiple histone loaders. Notably, the EBV tegument protein BNRF1 targets ATRX and DAXX for sequestration in promyelocytic leukemia (PML) bodies at this time point (24), suggesting that HIRA and newly induced CAF1 may be responsible. H3.1 and H3.3 levels remained stable at day 4 postinfection, a time point at which cells divide every 8 to 12 h (11–13). Interestingly, after the period of Burkitt-like hyperproliferation that extended roughly from day 3 to day 7 postinfection, H3.1 and H3.3 levels nearly doubled, even controlling for increases in EBV genome copy numbers over this interval. This result suggests that each type of histone 3 is loaded by host machinery onto newly synthesized episomes, despite short cell cycle times (Fig. 6D).

### CAF1 is important for deposition of repressive H3K9me3 and H3K27me3 heterochromatin marks.

CAF1 has important roles in host genome heterochromatin organization (64–66), in part through cross talk with deposition of repressive histone 3 lysine 9 and 27 trimethyl (H3K9me3 and H3K27me3) marks. For instance, in cell fate determination, depletion of CHAF1A reduces H3K27me3 levels at promoters of many genes associated with pluripotency (64). Deposition of H3K9me3 and H3K27me3 marks onto the EBV genome are important for silencing of the lytic and latency III programs (67–72). However, potential CAF1 roles in regulation of EBV genome repressive H3 marks have not been investigated.

**FIG 7:**
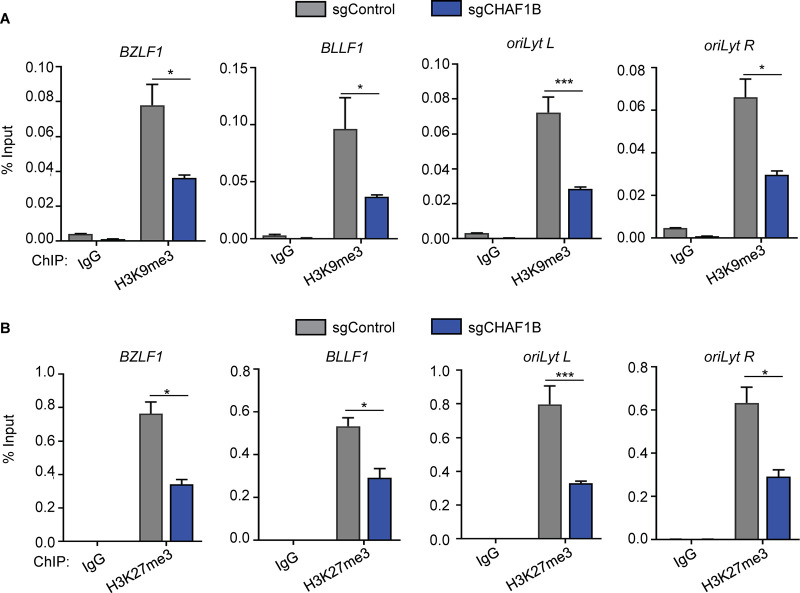
CHAF1b is important for H3K9me3 and H3k27me3 repressive marks at EBV genome lytic cycle regulatory sites. (A) ChIP for H3K9me3 was performed on chromatin from Akata EBV^+^ cells expressing control or CHAF1B sgRNA, followed by qPCR performed with primers specific for the *BZLF1* or *BLLF1* promoters, *oriLyt* R, or *oriLyt* L. Mean + SEM values are shown for *n* = 3 biologically independent replicates. *P* values were calculated by two-way ANOVA with Sidak’s multiple-comparison test. (B) ChIP for H3K27me3 was performed on chromatin from Akata EBV^+^ cells expressing control or CHAF1B sgRNAs, followed by qPCR performed with primers specific for the *BZLF1* or *BLLF1* promoters, *oriLyt* R, or *oriLyt* L. Mean + SEM values are shown for *n* = 3 biologically independent replicates. *P* values were calculated by two-way ANOVA with Sidak’s multiple-comparison test.

**FIG 8:**
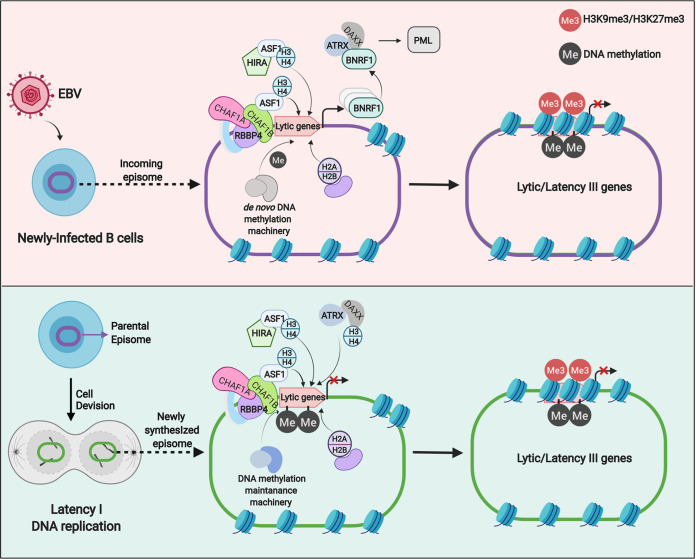
Schematic of histone loader roles in EBV genome regulation. (Top) CAF1 and HIRA load histones H3/H4 onto incoming EBV genomes, together with ASF1. H2A/H2B are loaded onto the EBV genome by distinct histone chaperones and assemble into histone octamers. EBV BNRF1 subverts ATRX/DAXX in newly infected cells. DNA methyltransferases and histone H3K9 and H3K27 methyltransferases add repressive marks that suppress lytic cycle and latency III genes. (Bottom) CAF1 and HIRA roles in maintenance of the latency I program. EBV genomes are replicated by host machinery in early S-phase, and newly synthesized genomes must be reprogrammed to latency I. CAF1, HIRA, and ATRX/DAXX have nonredundant roles in maintenance of latency I. Cross-talk with DNA methylation machinery is important for propagation of the CpG methylation marks that maintain latency I.

CRISPR knockout (KO) was used to test the effects of CHAF1B depletion on H3K9me3 and H3k27me3 marks at four EBV genomic sites known to carry these repressive marks in latency. Following expression of control versus CHAF1B sgRNAs in EBV^+^ Akata cells, ChIP was performed with control IgG or with antibodies against H3K9me3 or H3K27me3. qPCR analysis demonstrated that CHAF1B depletion significantly reduced H3K9me3 occupancy by 2-fold to 3-fold at the *BZLF1* and *BLLF1* promoters and at both *oriLyt* regions (Fig. 7A). Likewise, sgCHAF1B expression diminished levels of repressive H3K27me3 marks at the sites to a similar extent (Fig. 7B). These results are consistent with a key CAF1 role in H3.1 loading onto newly synthesized EBV DNA, which can then subsequently be modified by repressive histone H3K9me3 and H3K27me3 marks at appropriate viral genomic sites (Fig. 8).

We recently reported that host epigenetic enzyme UHRF1 and DNA methyltransferase 1 are important for maintenance of EBV latency in BL. UHRF1 domains that read H3K9me2/me3, the H3 N terminus, and hemimethylated DNA were each found to be essential for EBV latency I (73). We therefore speculated that CHAF1B depletion might also perturb UHRF1 recruitment and disrupt DNA methylation at key EBV genomic sites. To test these hypotheses, ChIP was first performed with control or anti-UHRF1 antibody on DNA from Akata cells expressing control or CHAF1B sgRNAs, followed by quantitative PCR (qPCR) for EBV regions of interest. CHAF1B depletion significantly reduced UHRF1 occupancy at the EBV W and C promoters (Fig. S8B), which are silenced in latency I but which drive the prelatency phase and latency III programs, respectively (43). Likewise, methyl DNA immunoprecipitation (MeDIP) and qPCR revealed that CHAF1B depletion reduced 5-methylcytosine levels at the EBV origins of lytic replication (*oriLyt R* and *L*) and at the late lytic gene *BLLF1*, despite the use of acyclovir to prevent the synthesis of unmethylated lytic EBV genomes (Fig. S8C).

## DISCUSSION

EBV coopts host epigenetic pathways to regulate viral genome programs. Incoming EBV genomes are organized into nucleosomes, which must then be maintained or remodeled on newly synthesized, damaged, or transcribed regions of EBV genomes. Burkitt lymphoma cells are among the fastest growing human tumor cells, and newly EBV-infected B-cells undergo Burkitt-like hyperproliferation between day 3 and day 7 postinfection in cell culture (11–13). Host machinery must therefore propagate chromatin-encoded epigenetic information with each cell cycle, a process which begins with histone loading. The results presented here suggest that EBV coopts the CAF1 complex to establish and maintain latency, reminiscent of its use by host pathways that regulate embryonic development and cell fate.

Histone H3 was loaded onto EBV genomes by 48 h after primary B-cell infection, by which time CAF1 expression was upregulated (36–39). These observations raise the issue of whether CAF1 participates in chromatin assembly on incoming viral genomes. While CRISPR technical limitations currently prevent us from addressing this issue with resting primary B-cells, results obtained with our Akata cell system suggested that CAF1 may play a key role in latency establishment. However, in contrast to newly infected primary cells, Akata cells rapidly divide, and our experiments did not differentiate between disruption of latency establishment and reactivation after the first cell cycle, perhaps related to defects in DNA replication-coupled histone loading.

It is plausible that other histone chaperones, in particular, HIRA, may play key roles in H3/H4 loading onto incoming EBV genomes prior to mitosis in newly infected cells. CAF1 may then carry out DNA replication-dependent roles, beginning with entry of the newly infected cell into S-phase at approximately 72 h postinfection (11–13), and HIRA plays ongoing roles that remain to be defined. Notably, EBV tegument protein BNRF1 subverts DAXX/ATRX-mediated H3.3 loading on viral chromatin for the first several days postinfection and presumably also with lytic reactivation (23, 24) (Fig. 8). ATRX is necessary for maintenance of EBV latency, suggesting complex interplay between multiple histone chaperones.

Histones 3.1 and 3.3 are loaded onto the betaherpesvirus cytomegalovirus genomes (53), and, intriguingly, their deposition did not require transcription or replication of the viral genome. This finding raises the possibility that conserved mechanisms may load histones onto herpesvirus genomes more broadly. Histone 3.1 and 3.3 loading regulates key aspects of herpes simplex virus gene regulation (52, 74, 75). Likewise, since CAF1 is a key histone 3.1/4 chaperone, its depletion may have both direct and indirect effects on viral genome regulation, including those that occur as a result of changes in host gene expression that secondarily affect the EBV genome.

Histone chaperone ASF1 transports H3/H4 complexes to the nucleus for deposition onto DNA by CAF1 or HIRA (76). While ASF1a preferentially associates with HIRA and ASF1b with CAF1, they can function redundantly when coexpressed. Depletion of both ASF1A and ASF1B is required to arrest human cell DNA replication (77). Since EBV B-cell infection upregulates both ASF1a and ASF1b transcripts (37), and since ASF1a and ASF1b are highly coexpressed in Burkitt cells (73), we speculate that this redundancy precluded either ASF paralogue from scoring in our latency reversal CRISPR screen (25). It is interesting that Tousled like kinases (TLK) have been implicated in the maintenance of gammaherpesvirus latency (78), and a major TLK function is to regulate ASF1 (79). The CHAF1B KO may have had stronger lytic induction phenotypes than targeting of other CAF-1 subunits because it functions as a central facilitator of CAF1 functions, including by mediating interactions between ASF1/H3/H4 and CHAF1A (80).

Further studies are required to identify how HIRA, ATRX, and DAXX maintain latency I but permit EBNA1 and EBV noncoding RNA expression. It also remains possible that they indirectly control EBV genome expression through effects associated with host transcription factor expression, which then secondarily regulate the EBV genome. Further underscoring the intricate relationship between EBV and histone biology, EBNA3C downregulates the histone H2A variant H2AX shortly after primary B-cell infection at the mRNA and protein levels (81).

MYC suppresses EBV reactivation by preventing DNA looping of *oriLyt* and terminal repeat regions to the *BZLF1* promoter (25). The results presented here raise the issue of whether histone loading by CAF1 or HIRA may act together with MYC to prevent long-range EBV genomic DNA interactions that promote lytic reactivation, perhaps at the level of CTCF, cohesion, or other DNA looping factors. Notably, MYC mRNA abundance was only modestly reduced by CHAF1B depletion (see Table S1 in the supplemental material). Perturbation of histone loading may alternatively be sufficient to derepress *BZLF1*. Indeed, micrococcal nuclease digestion experiments demonstrated that immediate early *BZLF1* and *BRLF1* promoters are nucleosomal (82). Yet, open chromatin at *BZLF1* and *BRLF1* is not sufficient for lytic reactivation (71). Furthermore, CHAF1B derepression strongly derepressed latency III gene expression, suggesting a broader role in silencing of EBV antigens.

We recently identified that the facilitated chromatin transcription (FACT) histone loading complex is critical for EBV Burkitt latency (25). FACT remodels histones at sites of active transcription to enable RNA polymerase processivity (83, 84). Further underscoring diverse histone chaperone roles in maintenance of EBV latency, FACT was found to regulate EBV latency through effects on *MYC* expression, consistent with its role in driving glioblastoma oncogenic *N-MYC* expression (85). However, we observed only modest reduction in *MYC* mRNA expression upon CHAF1B knockdown (Table 1), suggesting an alternative mechanism for its EBV latency maintenance role.

**TABLE 1:** CHAF1B KO and rescue^*a*^

Molecule	Sequence
sgRNA	5′-GCTGAACAAGGAGAACTGGA-3′ (sense)
Genomic DNA	5′-GCTGAACAAGGAGAACTGGAC***G***GT-3′ (#1)
Rescue cDNA	5′-GCTGAACAAGGAGAACTGGAC***A***GT-3′ (#1)
Rescue cDNA sequence surrounding the PAM site mutation	GGAGGATCCACAGACTGGCGTCTGCCGGCGTGGACACCAATGTCAGGATCTGGAAGGTAGAAAAGGGACCAGATGGAAAAGCCATCGTG GAATTTTTGTCCAATCTTGCTCGTCATACCAAAGCCGTCAATGTTGTGCGTTTTTCTCCAACTGGGGAAATTTTAGCATCGGGAGGAGATGA TGCTGTCATCCTATTGTGGAAGGTGAATGATAACAAGGAGCCGGAGCAGATCGCTTTTCAGGATGAGGACGAGGCCCAGCTGAACAAGG AGAACTGGAC*A*GTTGTGAAGACTCTGCGGGGCCACTTAGAAGATGTGTATGATATTTGCTGGGCAACTGATGGGAATTTAATGGCTTCTG CCTCTGTGGATAACACAGCCATCATATGGGATGTCAGCAAAGGACAAAAGATATCAATTTTTAATGAACATAAAAGTTATGTCCAAGGAG TAACCTGGGACCCTTTGGGTCAATATGTTGCTACTCTGAGCTGTGACAGGGTGCTGCGAGTATACAGTATACAGAAGAAGCGTGTGGCTTT CAATGTTTCGAAGATGCTGTCTGG

a The CHAF1B sg1 sequence is shown. PAM sequences are underlined. The PAM site mutation is indicated in bold and italic.

A longstanding issue has remained concerning how lytic EBV genomes destined for packaging into viral particles evade histone loading, since histones are not detectable in purified EBV viral particles (86). EBV lytic replication is initiated in early S-phase, taking place in nuclear factories that are devoid of histones or host DNA. CAF1 is recruited to host DNA replication forks through association with the DNA clamp PCNA. While PCNA can be detected in EBV amplification factories, it does not localize to sites of viral DNA synthesis (16). Abundances of CHAF1A, CHAF1B, ASF1a, and ASF1b decline in lytic replication in Burkitt/epithelial cell somatic hybrid D98/HR1 cells, whereas HIRA and DAXX levels were stable.

DNA methylation is important for suppression of latency III, raising the issue of how CHAF1B depletion derepressed latency III genes (see Fig. S3A in the supplemental material). While we note that latency III transcripts are induced by EBV lytic reactivation (28), we speculate that CAF1 may also repress latency III through cross talk with the enzyme UHRF1, which has key epigenetic roles in propagation of DNA methylation (67, 87, 88). CHAF1B depletion reduced UHRF1 recruitment to the viral W and C promoters and diminished EBV genomic CpG methylation, perhaps by reducing histone H3 and H3K9me2/3 levels. We recently reported that UHRF1 PHD and TTD domains, which recognize H3 and H3K9me2/3, respectively, are each important for propagation of EBV genomic CpG methylation (73).

CHAF1B depletion resulted in a strong interferon-induced signature, which we and others have not observed in Burkitt cell lytic reactivation triggered by immunoglobulin cross-linking or by conditional BZLF1 alleles. Therefore, we hypothesize that DNA sensing pathways may be activated by CHAF1B depletion, for example, in response to exposure of viral or host nonchromatinized DNA. Alternatively, latency III triggers expression of interferon-induced genes, and a derepressed EBV transcript may be responsible for this phenotype (36, 37, 87). It is also worth noting that CHAF1B depletion resulted in downregulation of numerous histone and histone-like genes, which we speculate may have resulted from the activity of a negative-feedback loop that responds to loss of this important histone chaperone complex.

EBV establishes lytic infection in normal, differentiated epithelial cells (88–91). Epithelial cell replication plays important roles in EBV shedding into saliva (92), and uncontrolled lytic EBV replication can cause oral hairy leukoplakia in heavily immunosuppressed people. It will be of significant interest to determine how the roles of CAF1, HIRA, ATRX, and DAXX in epithelial cells in supporting escape from EBV latency may be distinct.

Current Burkitt lymphoma therapies cause major side effects and increase the risk of secondary malignancies. Endemic BL management is further complicated by the risk of giving high-intensity chemotherapy in resource-limited settings. Consequently, there is significant interest in developing safer therapeutic regimens, including EBV lytic reactivation strategies (93). Reversal of EBV Burkitt latency could selectively sensitize tumor cells to T-cell responses and to the antiviral drug ganciclovir (94).

## MATERIALS AND METHODS

### Cell lines and culture.

Throughout the study, all B-cell lines used stably expressed S. pyogenes Cas9. The EBV^+^ Burkitt lymphoma cell lines P3HR-1, Akata, and MUTU I were used in the study. EBV^−^ Akata cells are a cell line derived from the original EBV^+^ Akata tumor cell line that spontaneously lost EBV in culture (55). The EBV^+^ Burkitt lymphoma cell lines Akata EBV^+^, MUTU I, and P3HR-1 and EBV^−^ Akata cells were maintained in RMPI 1640 (Gibco, Life Technologies) supplemented with 10% fetal bovine serum (Gibco). HEK-293 T cells were grown in Dulbecco’s modified Eagle’s medium (DMEM) with 10% fetal bovine serum (Gibco). Cell lines with stable expression of Streptococcus pyogenes Cas9 genes were generated by lentiviral transduction, followed by blasticidin selection at 5 μg/ml, as previously reported (95). For selection of transduced cells, puromycin was added at the concentration of 3 μg/ml. Hygromycin was used at 200 μg/ml for the initial 4 days and at 100 μg/ml thereafter. Acyclovir was used at the concentration of 100 μg/ml *in vitro*. EBV producer HEK-293 cells stably transformed by the use of a BART-repaired B95-8-based EBV BAC system encoding GFP (57) were cultured in RPMI 1640 (Gibco, Life Technologies) supplemented with 10% fetal bovine serum (Gibco), 1% penicillin, and 50 μg/ml hygromycin. The 2-2-3 EBNA2-HT LCL with a conditional EBNA2 allele was a kind gift from Bo Zhao and Elliott Kieff (Brigham & Women’s Hospital, Harvard Medical School). 2-2-3 LCLs express EBNA2 fused to a modified estrogen receptor 4HT-binding domain. The EBNA2-HT allele localizes to the nucleus and is active in the presence of 4HT but upon 4HT withdrawal is redistributed to the cytosol, where it is destabilized. 2-2-3 LCLs were maintained in the presence of 1 μM 4-hydroxytamoxifen (4HT). To remove 4HT, cells were washed five times with 4HT-free media, including two incubations for 30 min, and were then reseeded at 50,000 cells per ml in media with or without 4HT, as indicated. Cells were then grown for 48 h and harvested for cell lysate preparation. All cells used in this study were cultured in a humidified incubator at 37°C with 5% CO_2_ and routinely tested and certified mycoplasma-free using a MycoAlert kit (Lonza). Short tandem repeat (STR) analysis (Idexx) was done to verify the identity of MUTU I cells.

### Immunoblot analysis.

Immunoblot analysis was performed as previously described (96). In brief, whole-cell lysates (WCL) were separated by SDS-PAGE, transferred onto nitrocellulose membranes, blocked with 5% milk–TBST (Tris-buffered saline with Tween 20) buffer, probed with primary antibodies at 4°C overnight on a rocking platform, washed four times, and then incubated with secondary antibody (Cell Signaling Technology, catalog no. 7074 and catalog no. 7076) for 1 h at room temperature. Blots were then developed by incubation with ECL chemiluminescence for 1 min (Millipore, catalog no. WBLUF0500), and images were captured by the use of a Li-Cor Fc platform. All antibodies used in this study are listed in Table S2 in the supplemental material. Bands on Western blots were selected using same-sized rectangles, and their signal intensities were measured by using the Analysis module of Image Studio Lite Ver 5.2 (Li-Cor). The relative abundances of protein species were calculated by dividing the intensity of the viral protein band by the intensity of the GAPDH (glyceraldehyde-3-phosphate dehydrogenase) band.

10.1128/mBio.01063-20.10 TABLE S2 List of antibodies, cell lines, reagents, kits, and oligonucleotides used in this study. Download Table S2, DOCX file, 0.02 MB. Copyright © 2020 Zhang et al. 2020 Zhang et al. This content is distributed under the terms of the Creative Commons Attribution 4.0 International license.

### Flow cytometry analysis.

For staining of live cells, 1 × 10^6^ cells were washed twice with FACS buffer (phosphate-buffered saline [PBS], 1 mM EDTA, and 0.5% bovine serum albumin [BSA]), followed by incubation of primary antibodies for 30 min on ice. Labeled cells were then washed three times with FACS buffer. Data were recorded with a BD FACSCalibur cell analyzer and analyzed with FlowJo X software (FlowJo).

### Quantification of EBV genome copy number.

To measure EBV genome copy number, intracellular viral DNA and virion-associated DNA present in cell culture supernatants were quantitated by qPCR analysis. For intracellular viral DNA extraction, total DNA from 2 × 10^6^ Burkitt cells was extracted by the use of a Blood & Cell Culture DNA minikit (Qiagen catalog no. 13362). For extracellular viral DNA extraction, 500 μl of culture supernatant was collected from the same experiment as was used for the intracellular DNA measurement and was treated with 20 μl RQ1 DNase (Promega) for 1 h at 37°C to degrade nonencapsidated EBV genomes. A 30-μl volume of proteinase K (New England Biolabs, catalog no. P8107S) (20 mg/ml) and a 100-μl volume of 10% (wt/vol) SDS (Invitrogen, catalog no. 155553-035) were then were added to the reaction mixtures, which were incubated for 1 h at 65°C. DNA was purified by phenol-chloroform extraction followed by isopropanol-sodium acetate precipitation and then resuspended in 50 μl nuclease-free water (Thermo Fisher, catalog no. 10977-023). Extracted DNA was further diluted to 10 ng/μl and subjected to qPCR targeting of the EBV *BALF5* gene. Standard curves were made by serial dilution of pHAGE-BALF5 miniprep DNA at 25 ng/μl. The viral DNA copy number was calculated by inputting sample threshold cycle (*C_T_*) values into the regression equation dictated by the standard curve.

### cDNA rescue assay.

V5-tagged CHAF1B cDNA with a G360A PAM site mutation was synthesized by GenScript (Piscataway, NJ), as indicated in Table 1. Rescue cDNA was synthesized by GenScript (Piscataway, NJ) and cloned into pLX-TRC313 vector. Cas9-expressing B-cells with stable C-terminal V5 epitope-tagged CHAF1B cDNA expression were established by lentiviral transduction and hygromycin selection. The sequences are listed in Table 1.

### Chromatin immunoprecipitation (ChIP) qPCR.

Cells were cross-linked with formaldehyde 0.4% for 10 min at room temperature, and the reaction was stopped by adding glycine (2.5 M) for 10 min at room temperature to reach a final 0.2 M concentration. The cells were washed three times with PBS and then lysed by the use of 1% SDS lysis buffer (50 mM Tris [pH 8.1], 10 mM EDTA, 1% SDS, and protease inhibitor) for 20 min on ice. Lysate was sonicated for 25 min (30 s on/30 s off) in a Diagenode water bath-sonicator and centrifuged at 13,000 rpm for 10 min. The supernatant was diluted 1:10 in ChIP dilution buffer (SDS 0.01%, Triton X-100 1.1%, 1.2 mM EDTA [pH 8], 16.7 mM Tris-HCl [pH 8.1], 167 mM NaCl, and protease inhibitor) and precleared for 1 h with rotation at 4°C with blocking beads. Soluble chromatin was diluted and incubated with 4 μg anti-HA polyclonal antibody (Abcam, catalog no. ab9110), anti-H3.1/H3.2 polyclonal antibody (Millipore, catalog no. ABE154), anti-H3.3 polyclonal antibody (Millipore, catalog no. 09-838), or anti-UHRF1 (Diagenode, ChIP grade, catalog no. C15410258-100). Specific immunocomplexes were precipitated with protein A beads (Thermo Fisher, catalog no. 101041). The beads were washed, for 5 min, once in low-salt buffer (SDS 0.1%, Triton X-100 1%, 2 mM EDTA [pH 8.1], 20 mM Tris-HCl [pH 8.1], 150 mM NaCl), twice in high-salt buffer (SDS 0.1%, Triton X-100 1%, 2 mM EDTA [pH 8], 20 mM Tris-HCl [pH 8.1], 500 mM NaCl), once in LiCl buffer (0.25 M LiCl, NP-40 1%, sodium deoxycholate 1%, 1 mM EDTA [pH 8.1], 10 mM Tris-HCl [pH 8.1]), and twice in Tris-EDTA (TE) buffer. After reverse cross-linking, DNA was purified by using a QIAquick PCR purification kit (Qiagen, catalog no. 28106). qPCR was used to quantify the DNA from the ChIP assay and to normalize it to the percentage of input DNA. Primers for qPCR are listed in Table S2.

### RT-PCR analysis.

Total RNA was harvested from cells using an RNeasy minikit (Qiagen, catalog no. 27106). Genomic DNA was removed by using an RNase-Free DNase set (Qiagen, catalog no. 79254). RNA was subjected to reverse transcription by the use of iScript reverse transcription supermix (Bio-Rad, catalog no. 1708841). qRT-PCR was performed using Power SYBR green PCR mix (Applied Biosystems, catalog no. 4367659) on a CFX96 Touch real-time PCR detection system (Bio-Rad), and data were normalized to internal control GAPDH. Relative expression levels were calculated using the 2^−ΔΔ^*^CT^* method. All samples were run in technical triplicate, and at least three independent experiments were performed. The primer sequences were listed in Table S2.

### MeDIP assay.

Genomic DNA was purified using a Blood & Cell Culture DNA minikit and then subjected to MeDIP assay using a MagMeDIP kit (catalog no. C02010021; Diagenode Diagnostics). qPCR assays were then performed as described above. The primers for qPCR are listed in Table S2.

### Primary human B-cell purification.

Discarded, deidentified leukocyte fractions left over from platelet donations were obtained from the Brigham and Women’s Hospital blood bank. Peripheral blood cells were collected from platelet donors, following institutional guidelines. Since these were deidentified samples, the gender was unknown. Our studies on primary human blood cells were approved by the Brigham & Women’s Hospital Institutional Review Board. Primary human B-cells were isolated by negative selection using RosetteSep human B-cell enrichment and EasySep human B-cell enrichment kits (Stem Cell Technologies, catalog no. 15064 and catalog no. 19054), according to the manufacturer’s protocols. B-cell purity was confirmed by determination of plasma membrane CD19 positivity through FACS analysis. Cells were then cultured with RPMI 1640 with 10% fetal bovine serum (FBS).

### EBV infection of primary B-cells.

EBV B95-8 virus was produced from B95-8 cells with conditional ZTA expression. 4HT was used at a concentration of 1 μM to induce EBV lytic replication and removed 24 h later, and cells were resuspended in 4HT-free RPMI 1640–10% FBS for 96 h. Virus-containing supernatants were collected and subjected to filtration through a 0.45-μm-pore-size filter to remove producer cells. Titer was determined experimentally by transformation assay as described previously (36). For analysis of transforming EBV production in Burkitt knockout experiments, culture supernatants from Akata EBV^+^ cells expressing control, CHAF1B, or HIRA sgRNAs were harvested. The supernatants were passed through a 0.80-μm-pore-size filter to remove any producer cells and were then mixed with 1 million purified CD19^+^ primary human B-cells in 12-well plates. For determining histone H3.1 or H3.3 occupancy in newly infected primary cells, 6 × 10^7^ purified human B-cells were infected with B95.8 at an MOI of 0.2. Ten million cells were harvested at 2, 4, and 7 days postinfection (dpi). The viral episome number at each time point was quantitated by *BALF5* qPCR. Recombinant vector pHAGE-BALF5 was used to establish the standard curve for absolute quantification of EBV episome number. The H3 ChIP qPCR signals were normalized using EBV episome numbers at each time point, in order to control for changes in EBV copy number in B-cells between dpi 2 and 7.

### Cocultivation of Akata EBV-negative cells and EBV HEK-293 producer cells.

EBV producer HEK-293 cells stably infected by the use of a BART-repaired B95-8-based GFP-EBV BAC system (57) and 2-8-15 cells. EBV producer cells were seeded at a density of 0.3 × 10^6^/ml in a Corning BioCoat collagen I 6-well plate (catalog no. 356400). After 24 h, HEK-293 producer cells were cotransfected with 500 μg of pCDNA-BALF4 and 500 μg of pCDNA-BZLF1 per well, as described previously (97). After incubation was performed for an additional 48 h, 0.5 × 10^6^/ml of control or CHAF1B KO Akata EBV^−^ cells resuspended in fresh media were added onto the HEK-293 cells gently. Cocultured cells were then mock-treated or treated with 100 μg/ml acyclovir (catalog no. 114798; Millipore). After an additional 48 h, Akata cells were resuspended carefully, without disturbing the HEK-293 monolayer, transferred into a new 6-well plate, and further settled for another 24 h for the removal of potentially contaminating HEK-293 cells. Akata cells were then subjected to gp350 PM FACS analysis. Forward scatter (FSC) and side scatter (SSC) parameters were used to exclude any potentially contaminating HEK-293 producer cells.

### RNA sequencing (RNAseq) analysis.

Total RNAs were isolated using an RNeasy minikit and the manufacturer’s protocol. An in-column DNA digestion step was included to remove residual genomic DNA contamination. To construct indexed libraries, 1 μg of total RNA was used for poly(A) mRNA selection using a NEBNext poly(A) mRNA magnetic isolation module (New England Biolabs), followed by library construction via the use of a NEBNext Ultra RNA library prep kit for Illumina (New England Biolabs). Each experimental treatment was performed in triplicate. Libraries were multiply indexed, pooled, and sequenced on an Illumina NextSeq 500 sequencer using single-end 75-bp reads (Illumina).

For RNA-seq data analysis, paired-end reads were mapped to human (GENCODE v28) and Akata EBV genomes. Transcripts were quantified using Salmon v0.8.2 (98) using quasi-mapping and GC bias correction mode. A read count table of human and EBV genes was then normalized across compared cell lines/conditions, and differentially expressed genes were evaluated using DESeq2 v1.18.1 (99) under default settings.

Volcano plots were built based on Log_2_ (fold change) and −log_10_ (*P* value) data with GraphPad Prism7. Heat maps were generated by feeding the Z-score values of selected EBV genes from DESeq2 into Morpheus (https://software.broadinstitute.org/morpheus/). Enrichr was employed to perform gene list-based gene set enrichment analysis on selected gene subsets (100). Consistently enriched gene sets in top 5 terms ranked by Enrichr adjusted *P* values were visualized GraphPad Prism 7.

### Statistical analysis.

Data are presented as means ± standard errors of the means. Data were analyzed using analysis of variance (ANOVA) with Sidak’s multiple-comparison test or two-tailed paired Student's *t* test with Prism7 software. For all statistical tests, a *P* cutoff value of <0.05 was used to indicate significance.

### Data availability.

RNAseq data were deposited in the NIH GEO database under accession no. GSE148910.
