# Supplementary material for: Histone Loaders CAF1 and HIRA Restrict Epstein-Barr Virus B-Cell Lytic Reactivation
Source: mBio. 2020 Oct 27;11(5):e01063-20. doi: 10.1128/mBio.01063-20 (PMC7593962; doi:10.1128/mBio.01063-20)
Supplement: TABLE S2 [file mBio.01063-20-st002.docx]

**Supplementary Table S2 List of antibodies, cell lines, reagents, kits, and oligoes used in this study**

| **REAGENT or RESOURCE** | **SOURCE** | **IDENTIFIER** |
| --- | --- | --- |
| **Antibodies** | | |
| Anti-GAPDH rabbit monoclonal antibody | Cell Signaling Technology | 5174S |
| Anti-chaf1b rabbit polyclonal antibody | Proteintech | 27633-1-AP |
| Anti-chaf1a rabbit polyclonal antibody | Proteintech | 17037-1-AP |
| Anti-RBBP4 rabbit polyclonal antibody | Proteintech | 20364-1-AP |
| Anti-HIRA rabbit polyclonal antibody | Cell Signaling Technology | 12463 |
| Anti-ATRX rabbit monoclonal antibody | Cell Signaling Technology | 14820 |
| Anti-DAXX rabbit monoclonal antibody | Cell Signaling Technology | 4533 |
| Anti-BZLF1 mouse monoclonal antibody | Santa Cruz Biotechnology | sc-53904 |
| Anti-EBVgP350 (72A1) mouse monoclonal antibody | BioXCell | N/A |
| Anti-BMRF1 (OT14E2) mouse monoclonal antibody | A gift from Richard Longnecker | N/A |
| Anti-Mouse IgG, HRP-coupled secondary antibody | Cell Signaling Technology | 7076 |
| Anti-Rabbit IgG, HRP-coupled secondary antibody | Cell Signaling Technology | 7074 |
| Anti-H3.1/H3.2 Rabbit monoclonal antibody | Millipore | ABE154 |
| Anti-H3.3 Rabbit monoclonal antibody | Millipore | 09-838 |
| Anti-HA Rabbit monoclonal antibody | Abcam | ab9110 |
| Anti-DDX1 Rabbit polyclonal antibody | Bethyl Laboratories | A300-521A |
| Anti-UHRF1 Rabbit polyclonal antibody | diagenode | C15410258 |
| **Recombinant DNA** | | |
| Avana CRISPR Lentivirus Library | Broad Institute |  |
| pLentiGuide-Puro | Addgene | 52963 |
| pLenti SpBsmBI sgRNA Hygro | Addgene | 62205 |
| pXPR-011 | Addgene | 59702 |
| pLX-TRC313 | Broad Institute |  |
| pCDNA-BALF4 | gift from Dr. Teru Kanda | N/A |
| pCDNA-BZLF1 | Gift from Dr.Teru Kanda | N/A |
| **Assay kits** | | |
| QiAquick PCR Purification Kit | Qiagen | 28106 |
| Blood & Cell Culture DNA Maxi Kit | Qiagen | 13362 |
| QIAprep Spin Miniprep Kit | Qiagen | 27106 |
| Invitrogen PureLink Quick Plasmid Maxiprep Kit | Invitrogen | K210016 |
| DNeasy Blood& Tissue Kit | Qiagen | 69504 |
| QIAquick Gel Extraction Kit | Qiagen | 28704 |
| iScriptTM Reverse Transcription Supermix | Bio-Rad | 1708841 |
| Power SYBR Green PCR Master Mix | Applied Biosystems | 4367659 |
| Gateway™ LR Clonase™ II Enzyme Mix | Invitrogen | 11789-020 |
| NEBNext® Poly(A) mRNA Magnetic Isolation Module | New England Biolabs | E7490S |
| NEBNext® Ultra™ II Directional RNA Library Prep with Sample Purification Beads | New England Biolabs | E7765S |
| NEBNext® Multiplex Oligos for Illumina® (Index Primers Set 2) | New England Biolabs | E7500S |
| NEBNext® Multiplex Oligos for Illumina® (Index Primers Set 1) | New England Biolabs | E7335S |
| MagMeDIP qPCR Kit | diagnode | C02010021 |
| RossetteSep Human B cells | STEMCELL | 15064 |
| EasySep Negative Human B cell | STEMCELL | 19054 |
| Reagents | | |
| protein A beads | Thermo Scientific | 101041 |
| polybrene | Sigma-Aldrich | TR-1003-G |
| UltraPure 10%SDS | Invitrogen | 155553-035 |
| 0.5M EDTA pH8.0 | Invitrogen | 15575-038 |
| Proteinase K | New England Biolabs | P8107S |
| **Cell lines** | | |
| P3HR1 clone 16 | Dr. Elliott Kieff | N/A |
| EBV+ Burkitt lymphoma AKATA | Dr. Elliott Kieff | N/A |
| EBV+ Burkitt lymphoma Daudi cell line | ATCC | CCL-213 |
| EBV+ Burkitt lymphoma  P3HR1-Cas9 | Ma et al., 2017 | N/A |
| 293T | ATCC | CRL-3216 |
| EBV+ Burkitt lymphoma  AKATA-Cas9 | Guo et al., 2020 | N/A |
| EBV- Burkitt lymphoma  AKATA-cas9 | Guo et al., 2020 | N/A |
| **Software** | | |
| STAR2.5.2b | Dobin et al., 2013 | https://github.com/alexdobin/STAR |
| DESeq2 v1.14.1 | Love et al., 2014 | https://bioconductor.org/packages/release/bioc/html/DESeq2.html |
| GSEA | Subramanian et al., 2005 | http://software.broadinstitute.org/gsea/index.jsp |
| GraphPad Prism 7 | GraphPad Software | https://www.graphpad.com/scientific-software/prism/ |
| Flowjo X | Flowjo LLC. | <https://www.flowjo.com/> |
| **sgRNAs** | | |
| CHAF1B-1 sense | cccagGCTGAACAAGGAGAACTGGA | |
| CHAF1B-1 antisense | ﻿aaacTCCAGTTCTCCTTGTTCAGCc | |
| CHAF1B-2 sense | cccagAGACTGAGTTTCACTCCCGA | |
| CHAF1B-2 antisense | ﻿aaacTCGGGAGTGAAACTCAGTCTc | |
| CHAF1A-1 sense | cccagGGAGGCCAAGAAGAAGAAGG | |
| CHAF1A-1 antisense | ﻿aaacCCTTCTTCTTCTTGGCCTCCc | |
| CHAF1A-2 sense | cccagCTCGGCGTCGGACCGCTTTC | |
| CHAF1A-2 antisense | aaacGAAAGCGGTCCGACGCCGAGc | |
| RBBP4-1 sense | cccagGCATTCATCGACTTGTCCTG | |
| RBBP4-1 antisense | aaacCAGGACAAGTCGATGAATGCc | |
| RBBP4-2 sense | cccagATTGCCGTTACAGACCAGAA | |
| RBBP4-2 antisense | aaacTTCTGGTCTGTAACGGCAATc | |
| HIRA-1 sense | cccagGCCCAGATCATCGAACGGGA | |
| HIRA-1 antisense | aaacTCCCGTTCGATGATCTGGGCc | |
| HIRA-2 sense | cccagTGTGTGCGGTGGTCAAACAG | |
| HIRA-2 antisense | aaacCTGTTTGACCACCGCACACAc | |
| ATRX-1 sense | cccagGAGTTCAGTTGATCATCAAG | |
| ATRX-1 antisense | aaacCTTGATGATCAACTGAACTCc | |
| ATRX-2 sense | cccagCAGGATCGTCACGATCAAAG | |
| ATRX-2 antisense | aaacCTTTGATCGTGACGATCCTGc | |
| DAXX-1 sense | cccagCGGCGGAGTTCTGCAACATC | |
| DAXX-1 antisense | aaacGATGTTGCAGAACTCCGCCGc | |
| DAXX-1 sense | cccagCAAGGACCCGTGGTTCCCGG | |
| DAXX-1 antisense | aaacCCGGGAACCACGGGTCCTTGc | |
| MYC-1 sense | caccgGGTAGGGGAAGACCACCGAG | |
| MYC-1 antisense | aaacCTCGGTGGTCTTCCCCTACCc | |
| MYC-2 sense | caccgGTATTTCTACTGCGACGAGG | |
| MYC-2 antisense | aaacCCTCGTCGCAGTAGAAATACc | |
| **qPCR primers** | | |
| OrilytR_F | CGCTGGTTAAGCTGACGACCT | |
| OrilytR_R | GCCCTGGCTAGGAAAGGGAGGAA | |
| OrilytL_F | CCCTCAGTGTTCGCCAGCTT | |
| OrilytL_R | AGGGTGGGAGCAATTCCAACAC | |
| BALF5_F | GAGCGATCTTGGCAATCTCT | |
| BALF5_R | TGGTCATGGATCTGCTAAACC | |
| GAPDH_F | GTCTCCTCTGACTTCAACAGCG | |
| GAPDH_R | ACCACCCTGTTGCTGTAGCCAA | |
| BZLF1_F | AGGCCAGCTAACTGCCTATC | |
| BZLF1_R | TGATTCTGGGTTATGTCGGA | |
| BRLF1_F | ACACTCCCGGCTGTAAATTC | |
| BRLF1_R | TGGCTTGGAAGACTTTCTGA | |
| BLLF1_F | TGGCGAGTTTGCGTCCTCAG | |
| BLLF1_R | CGTCCAGTGTCACGATTTCTTGG | |
| BSLF1_F | GCGGGTCCTCTGGATTAGATAGTC | |
| BSLF1_R | CAGGGGCGGTGGTCTTAGC | |
| BDLF1_F | GCACCTCCTCTGCTATGGGC | |
| BDLF1_R | TGATACTCACCAAGATTGTTCCAGG | |
| BBLF4_F | AAGCCTGCCTCATCCTTGACC | |
| BBLF4_R | GACGAGCCTCTCCTTCACGG | |
| BMRF1_F | CGTGCCAATCTTGAGGTTTT | |
| BMRF1_R | CGGAGGCGTGGTTAAATAAA | |
| Cp_F | GGCGGGAGAAGGAATAACG | |
| Cp_R | CTTGAGCTCTCTTATTGGCTATAATCC | |
| Wp_F | AGTGGGCTTGTTTGTGACT | |
| Wp_R | TGACAATTGGCTGCTGTCT | |
